# Genotype by light quality interaction on the growth and development of quinoa (*Chenopodium quinoa*) and the crop response to salinity

**DOI:** 10.3389/fpls.2026.1725934

**Published:** 2026-06-12

**Authors:** Milton Gordillo-Romero, María de Lourdes Torres, Viviana Jaramillo Román, Daniel Dávila, Renato Morales-Acuña, Andrés F. Torres, Sofia D. Carvalho

**Affiliations:** 1 Laboratorio de Biotecnología Vegetal, Colegio de Ciencias Biológicas y Ambientales, Universidad San Francisco de Quito USFQ, Quito, Ecuador; 2 Radicle Crops, Wageningen, Netherlands; 3 Independent researcher, Lisbon, Portugal

**Keywords:** crop breeding, photobiology, physiology, quinoa, salinity

## Abstract

Quinoa (*Chenopodium quinoa* Willd.) is a highly nutritious crop with remarkable tolerance to drought and salinity, making it a promising model for developing resilient crops. In model plant species, photoreceptors have been shown to mediate abiotic stress responses. Although quinoa’s tolerance mechanisms have been extensively investigated, the influence of light on its growth and stress responses remains largely unexplored. This study evaluated the effects of broad-spectrum white light supplemented with narrow-bandwidth light on quinoa vegetative development under non-saline and moderate salinity conditions. Four genotypes were tested: two commercial varieties and two local Ecuadorian landraces. Light quality influenced physiological responses in a genotype-dependent manner. Supplemental far-red light promoted greater plant height under both control and salt conditions. Supplemental red light enhanced biomass accumulation under control conditions but not under salinity, suggesting that red light–mediated growth promotion may be constrained by higher salinity. Conversely, supplemental blue light mitigated the negative effects of salinity on growth, indicating a potential role as a positive mediator for salt tolerance in the species. The presence of salt altered several light-driven responses, supporting an interaction between responses to light and salt stress signaling. Our findings highlight the importance of incorporating light-related variables into quinoa stress physiology research which could ultimately provide breeding insights to enhance crop resilience through targeted photoreceptor breeding.

## 1 Introduction

Quinoa (*Chenopodium quinoa* Willd) is a nutritious seed crop native to the Andes, from southern Colombia to central Chile and Argentina (Jacobsen, 2003). This versatile crop demonstrates adaptability to diverse environments, thriving at altitudes ranging from sea level, including the arid mountainous and subtropical lowlands in Chile, to 4000 meters above sea level in the Peruvian and Bolivian Altiplano (Gomez-Pando et al., 2019). Its outstanding nutritional composition, rich in proteins, vitamins, and antioxidants, has earned it the title of “Golden grain of the Andes” (Mota et al., 2016; Nowak et al., 2016; Angeli et al., 2020), making it highly valued by farmers in regions facing food security challenges (Bazile et al., 2015). The germplasm of quinoa showcases remarkable diversity, with numerous ecotypes and varieties able to grow under different environmental conditions, including drought, poor soils, high salinity, and different photoperiods (De Santis et al., 2016; Gomez-Pando et al., 2019). Despite quinoa great adaptability and potential for the expansion of its cultivated area on a global scale, most of its production is still concentrated in the Andean countries (Alandia et al., 2020; Taaime et al., 2023).

Light regulates plant growth, physiological development, flowering time, and responses to stress (Roeber et al., 2021; Wei et al., 2023). Photoreceptors act as light sensors and detect specific wavelengths that trigger internal pathways regulating plant structure, physiology, and metabolism (Kami et al., 2010; Lamers et al., 2020; Paradiso and Proietti, 2022). Cryptochromes, for example, regulate drought stress-related genes in *Brassica napus* (Sharma et al., 2014), whereas phytochromes modulate salt and drought stress responses in tomato (*Solanum lycopersicum*) and rice (*Oryza sativa*) (D’Amico-Damião et al., 2015; Gavassi et al., 2017; Yoo et al., 2017). Light plays as well a critical role in plant homeostasis through photosynthesis, providing energy and governing essential metabolic functions (Feng et al., 2016). Although there is extensive research on the regulatory effects of light and their relationship with stress responses in model plants such as Arabidopsis, research and applications in crops like quinoa remain scarce (Indorf et al., 2007; Datta et al., 2008).

Given quinoa’s remarkable ability to tolerate diverse abiotic stresses, it represents a promising model for elucidating how light quality modulates stress response mechanisms in crops. This is particularly relevant for improving food production in increasingly challenging environments. Quinoa breeding programs are underway worldwide, with the aim of developing locally adapted cultivars and enhancing productive cropping systems (Murphy et al., 2018). Primary breeding targets include increased seed yield, seed quality, and plant resistance to biotic and abiotic stress (Murphy et al., 2018). Understanding the role of light in quinoa growth, development, and responses to stress may reveal new avenues to manipulate physiology and enhance crop resilience through genetic improvement and environmental management (Jiao et al., 2007; Yang et al., 2019). The goal of this study was to provide an initial assessment of the effects of light quality on the growth and development of quinoa under the absence and presence of salt. We analyzed four different quinoa genotypes, two commercial varieties and two local Ecuadorian landraces. Our results show that light quality influences quinoa development in a genotype-dependent manner. The presence of salt modulates plant responses to light. Differential light regimes represent a valuable tool for investigating the physiological and metabolic mechanisms underlying stress adaptation in quinoa. Such insights may be extended to other stress conditions, such as drought, and heat, and applied in breeding programs to enhance stress tolerance in quinoa.

## 2 Materials and methods

### 2.1 Plant material

We used seeds from the commercial varieties Bastille (Radicle Crops, The Netherlands) and Tunkahuan (Instituto Nacional de Investigaciones Agropecuarias, INIAP, Ecuador), as well as two landraces from the Ecuadorian highlands, 5_Chimborazo and 1_Cañar (Salazar et al., 2019). Bastille is a European early-flowering variety adapted to long-day photoperiods and representative of the Coastal ecotype, requiring approximately 110 days (16 weeks) from sowing to harvest under field conditions in Northern Europe. Due to its photoperiod sensitivity, Bastille completes its life cycle considerably faster under the short-day conditions of Ecuador, where it requires around 90 days (13 weeks) for complete senescence. In contrast, Tunkahuan is a late-flowering, short-day–adapted variety representative of the Inter-Andean Valley ecotype and requires roughly 170 days (24 weeks) from sowing to harvest under Ecuadorian field conditions. The landraces 5_Chimborazo and 1_Cañar also belong to the Inter-Andean Valley ecotype and are late-flowering materials adapted to short-day photoperiods, with growth cycles of approximately 180 days (26 weeks) under Ecuadorian field conditions. These landraces were selected for their contrasting salt-stress responses previously documented by (Jaramillo Roman, 2021). Under greenhouse conditions, they exhibited distinct physiological and agronomic responses to salinity: based on seed yield, the salt tolerance index of 1_Cañar (0.7) was higher than that of 5_Chimborazo (0.5), and while the harvest index of 1_Cañar remained stable under salt stress, 5_Chimborazo showed a 20% reduction. Together, the four genotypes represent a deliberate combination of contrast in phenological earliness (Bastille vs. Tunkahuan/1_Cañar/5_Chimborazo) and salinity-tolerance contrast (1_Cañar vs. 5_Chimborazo/Bastille/Tunkahuan). This design enables a more mechanistic evaluation of how light quality influences early-stage growth and development of quinoa under the absence and presence of salt.

### 2.2 Light conditions

Light was provided by ELIXIA light units (Heliospectra, Sweden). Assays were conducted inside four isolated cubicles separated by blackout curtains. Four combinations of light were used: control white; white + supplemental blue (450 nm); white + supplemental red (660 nm); and white + supplemental far-red (735 nm) (**Supplementary Figure 1**). Light intensity was set at 200 μmol m^-2^s^-1^ for the white control and at 300 μmol m^-2^s^-1^ (200 μmol m^-2^s^-1^ white plus 100 μmol m^-2^s^-1^ supplemental light) for the other three conditions (**Supplementary Figure 1B**). Photoperiod for all light treatments was 16h light/8h dark, and the temperature of the growing room was set at 21 °C. The distance between the light units and the plant canopy was initially adjusted to ensure uniform light distribution across the growing area (target setpoint +/- 10%). As plants grew, distance was adjusted as required to ensure light intensity at the top of the canopy remained constant. All plants were germinated and initially grown under white light. At Week 4 (transition point from seed germination and early seedling establishment to vegetative development) plants were moved to the different light treatments and grown for eight more weeks. Plant growth was assessed at Week 12, one week before expected life cycle ending of the fastest landrace, Bastille, as described in Section 1.2.

### 2.3 Control and salt treatments

A control without salt (salt control conditions) and a treatment group exposed to salt were evaluated under each of the four light treatments. Salt control groups consisted of four replicates of the four quinoa genotypes irrigated twice a week with 150 ml of a 1/2 Hoagland solution and no salt (0 mM NaCl). Salt-treated groups consisted of four replicates of the four quinoa genotypes irrigated twice per week with 150 ml of a 1/2 Hoagland solution supplemented with 200 mM NaCl. Salt accumulation in the substrate of salt treatment groups was monitored weekly by measuring the electrical conductivity (EC) of drained water and ensuring it did not exceed 20–30 miliSiemens/cm, which corresponds to the EC of a 200 mM NaCl solution. Saline irrigation of the treatment groups started at Week 6 and was maintained until the end of the experiment, at Week 12.

### 2.4 Experimental design

A split plot row-column experimental design was implemented (**Supplementary Figure 2**). Within each light treatment both a salt control group (0 mM NaCl) and a salt treatment group (200 mM NaCl) were included. Within each control/salt group the four quinoa genotypes were evaluated with four replicates per genotype. To minimize positional effects related to light uniformity, the spatial location of replicates was randomized using the split-plot row-column structure, allowing for spatial correction during statistical analysis with the StatgenSTA v1.0.15 R package (Bart-Jan, 2025).

### 2.5 Assessment of growth traits

All plants were photographed weekly and plant height was calculated by processing images with ImageJ (Schneider et al., 2012). Total biomass (dry weight) was determined at harvest (Week 12) after oven-drying the plants at 60 °C for 72 hours. To quantify biomass distribution, stems, leaves, and panicles were weighed separately. Total biomass is reported in grams of dry matter. At the end of the experiment (Week 12) relative growth rate (RGR, *d^−1^*) and its specific components were calculated based on the linear relation *RGR* = *LWR* × *SLA* × *NAR*, where NAR is the net assimilation rate (*g* x *m^−2^* x *d^−1^*), LWR is leaf weight ratio (*g* x *g^−1^*), and SLA is the specific leaf area (*m^2^* x *kg^−1^*). SLA was calculated as the amount of leaf area per unit of leaf dry weight, LWR as the leaf fraction of total dry plant biomass, and RGR as the natural logarithm of the relative increase in plant biomass over a set period of time: RGR = *(ln(W_2_) − ln(W_1_))/(t_2_ − t_1_)*.

### 2.6 Statistical analyses

Inferential statistical analyses were conducted to compare means among treatments. Depending on data distribution and variance homogeneity, either analysis of variance (ANOVA) or the non-parametric Kruskal–Wallis test were applied to evaluate the effects of light quality, salinity (200 mM NaCl), genotype, and their interactions (p < 0.05). Multiple comparison analyses were conducted using Tukey’s HSD test on genotype means using the Agricolae R package v1.3.7 (Mendiburu, 2023). Spatial correction of the data was performed using the StatgenHTP v1.0.9.1 (Millet and van Rossum, 2025). All statistical analyses were performed using the StatgenSTA v1.0.15 (Single Trial Analysis) R package (Bart-Jan, 2025).

## 3 Results

### 3.1 Physiological responses of four quinoa genotypes to broad white light supplemented with narrow-bandwidth light

#### 3.1.1 Plant height

**Figure 1:**
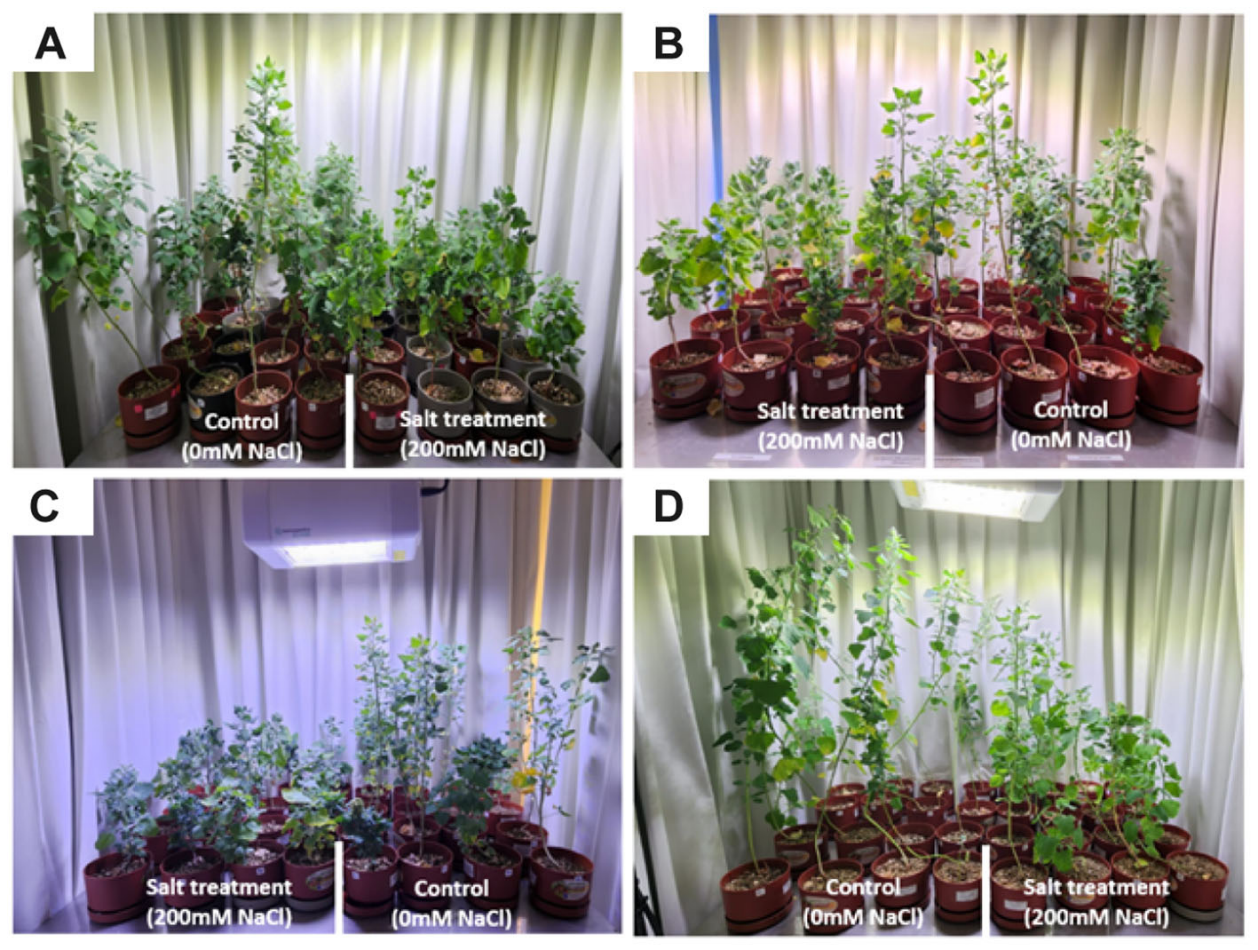
Photographic record of the quinoa plants growing under the four light treatments. **(A)** White light. **(B)** Red light. **(C)** Blue light, and **(D)** Far-red light at week 12.

**Figure 2:**
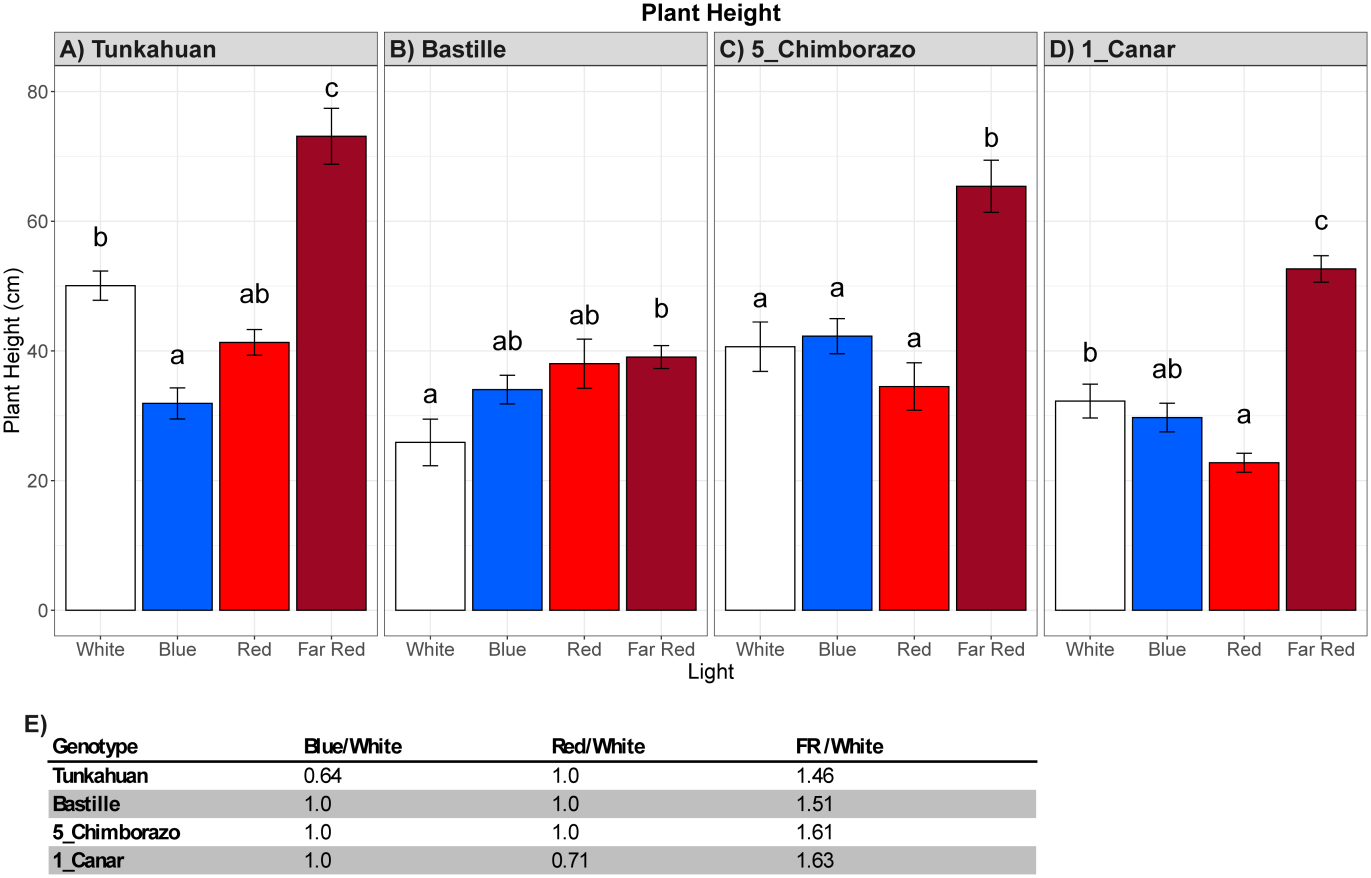
Plant height per genotype under four light treatments. **(A)** Tunkahuan, **(B)** Bastille, **(C)** 5_Chimborazo, **(D)** 1_Cañar. **(E)** Ratios of plant height under blue, red or far-red versus white control conditions; when differences between light treatments and white control were not significant for each genotype then ratios are equal to 1.0; when differences were statistically different the calculated ratios are shown. Different letters indicate significantly different values after ANOVA and Tukey tests. (p values: Tunkahuan 0.003, Bastille 0.033, 5_Chimborazo <0.001 and 1_Cañar <0.001).

We first analyzed the effect of the four light treatments on plant height across the four quinoa genotypes (**Figure 1**). Under white light, Tunkahuan and 5_Chimborazo were the tallest genotypes, with 50.0 cm and 40.7 cm, respectively. 1_Cañar showed intermediate height (32.3 cm) and Bastille elongated the least, reaching a final height of 25.9 cm (**Supplementary Figure 3A**). Under supplemental light, the four genotypes responded differently to the different light conditions. The most pronounced effect on plant height was observed under supplemental far-red light, where all genotypes exhibited a statistically significant increase in stem elongation compared to the white light control (**Figure 2**). Plant height increased in Tunkahuan, Bastille, 5_Chimborazo, and 1_Cañar by 46%, 51%, 61%, and 63%, respectively (**Figure 2E**). Supplemental blue and red light reduced plant height only in specific genotypes. Under blue light, Tunkahuan reached 64% of the height observed under white light (**Figures 2A, E**), whereas the other three genotypes were unaffected (**Figures 2B–E**). Under red light, only 1_Cañar elongated differently than under white light, achieving 71% of the height compared to the control (**Figures 2D, E**).

#### 3.1.2 Total biomass

**Figure 3:**
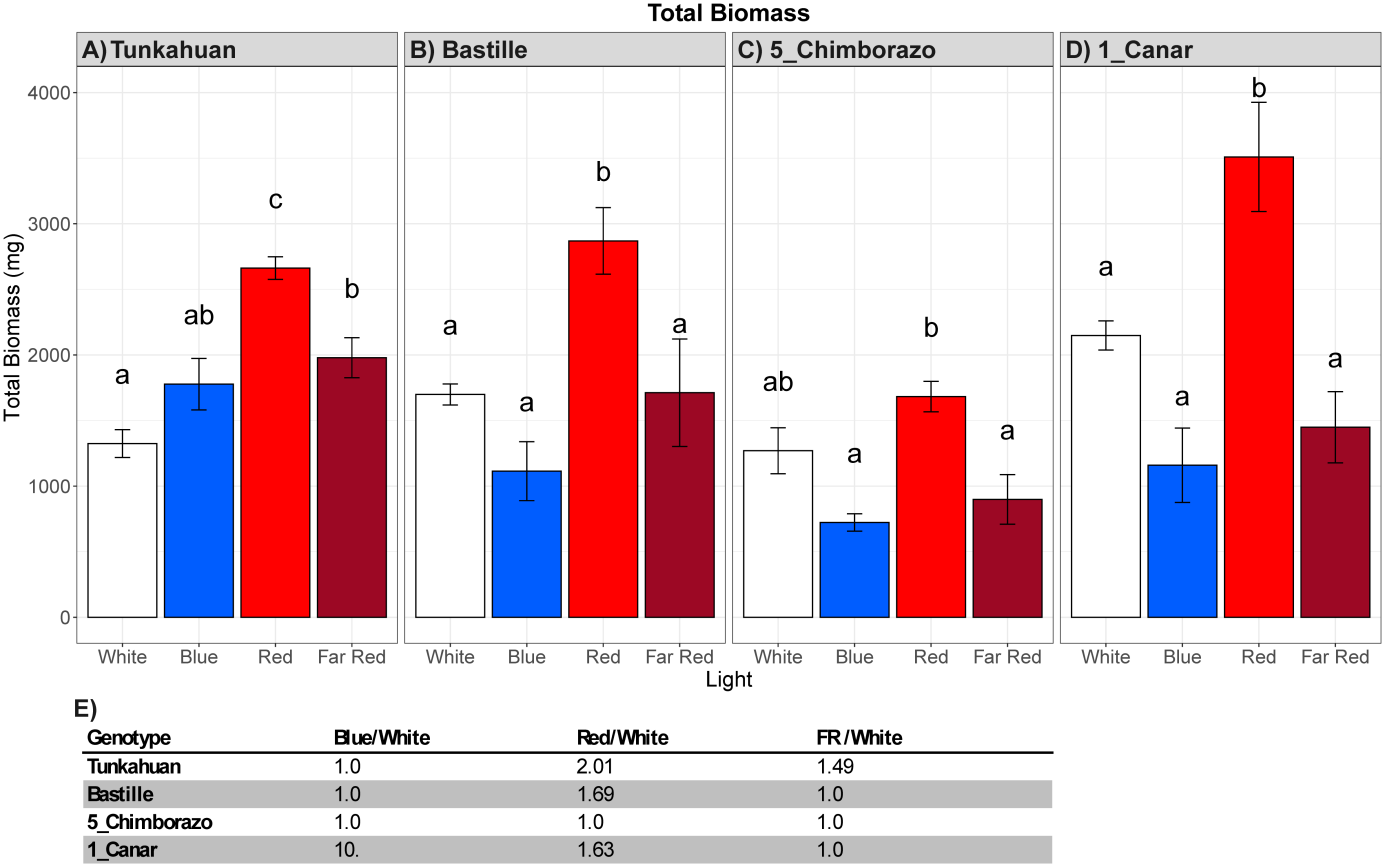
Total biomass per genotype under four light treatments. **(A)** Tunkahuan, **(B)** Bastille, **(C)** 5_Chimborazo, **(D)** 1_Cañar. **(E)** Ratios of total biomass under blue, red or far-red versus white control; when differences between light treatments and white control were not significant for each genotype then ratios are equal to 1.0; when differences were statistically different the calculated ratios are shown. Different letters indicate significantly different values after ANOVA and Tukey tests. (p values: Tunkahuan <0.001, Bastille 0.002, 5_Chimborazo 0.12 and 1_Cañar <0.001).

We next analyzed biomass production (mg/plant dry matter - DM) under the four light treatments (**Figure 3**; **Supplementary Figure 3B**). Under white light, 1_Cañar (2148 mg/plant DM) and Bastille (1698 mg/plant DM) showed the highest biomass, whereas biomass was lower for 5_Chimborazo and Tunkahuan with 1270 mg/plant DM and 1324 mg/plant DM, respectively (**Supplementary Figure 3B**). The three supplemental light conditions caused different effects on total biomass compared to the white control, with genotype-specific responses. The most pronounced effect was seen under supplemental red light, where biomass significantly increased in Tunkahuan by 101%, in Bastille by 69%, and in 1_Cañar by 63%, whereas 5_Chimborazo was unaffected (**Figure 3A-D**). Far-red supplementation only affected biomass of Tunkahuan, with a 49% increase compared to the control (**Figure 3A, E**). Supplemental blue light did not significantly affect biomass in any of the four genotypes compared to white light conditions (**Figure 3E**).

#### 3.1.3 Specific leaf are

**Figure 4:**
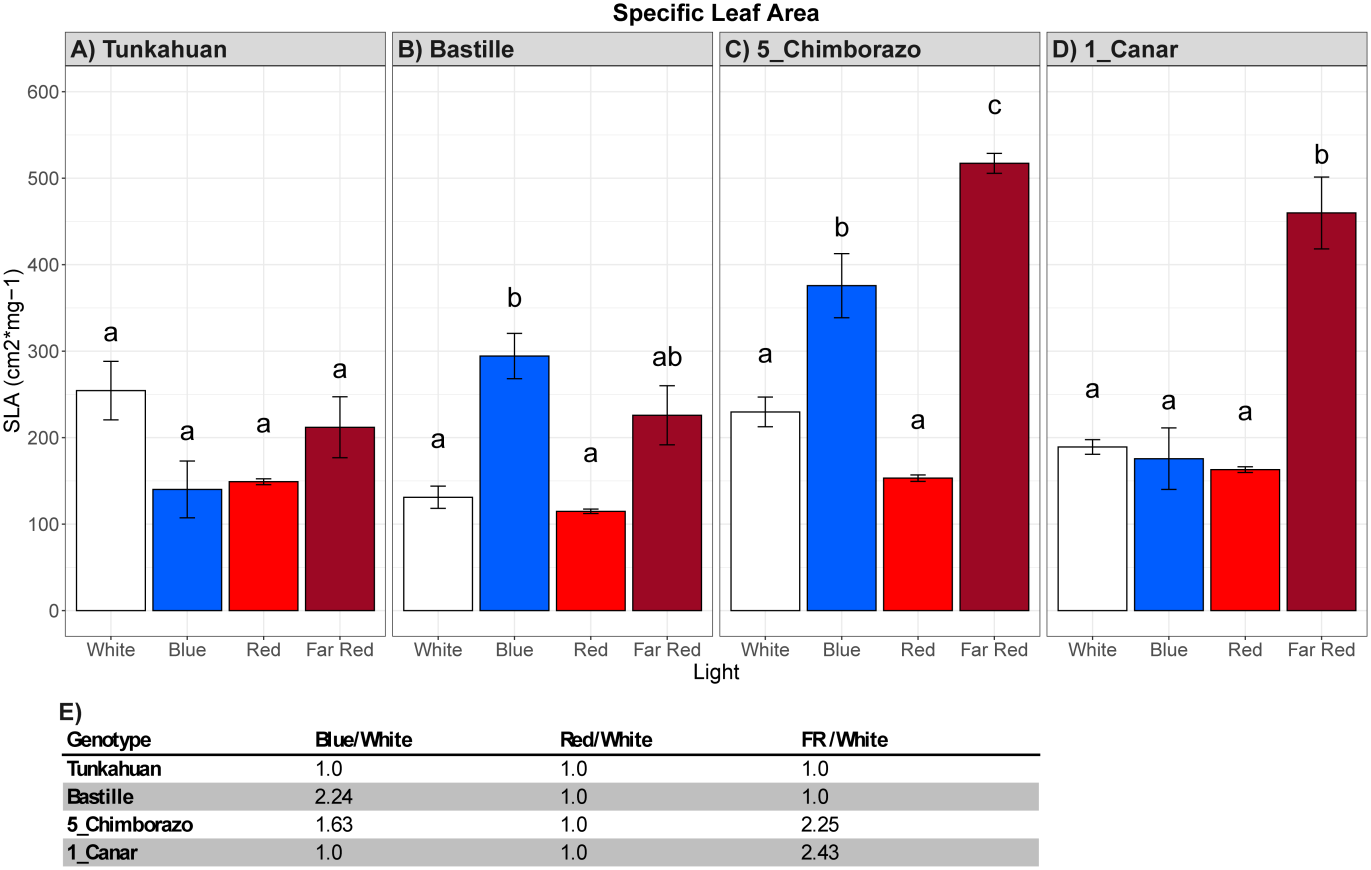
Specific leaf area (SLA) per genotype under four light treatments. **(A)** Tunkahuan, **(B)** Bastille, **(C)** 5_Chimborazo, **(D)** 1_Cañar. **(E)** Ratios of SLA under blue, red or far-red versus white control conditions; when differences between light treatments and white control were not significant for each genotype then ratios are equal to 1.0; when differences were statistically different the calculated ratios are shown. Different letters indicate significantly different values after ANOVA and Tukey tests. (p values: Tunkahuan 0.076, Bastille 0.002, 5_Chimborazo <0.001 and 1_Cañar <0.001).

The next physiological parameter measured was Specific Leaf Area (SLA, cm^2^ mg^-1^ –DM). Under white light, Tunkahuan exhibited the highest SLA (254 cm^2^ mg^-1^) while Bastille showed the lowest SLA (131 cm^2^ mg^-1^) – **Supplementary Figure 3C**. Under supplemental blue and far-red light, the four genotypes displayed contrasting responses (**Figure 4**). Under blue light, Bastille and 5_Chimborazo exhibited significant increase in SLA by 124% and 63%, respectively, compared to white light (**Figure 4B, C, E**). Under supplemental far-red light, SLA significantly increased by 125% in _Chimborazo and 143% in 1_Cañar, while remaining similar in Tunkahuan and Bastille compared to the control light treatment (**Figure 4C–E**). Supplemental red light did not alter the SLA in any of the four genotypes (**Figures 4A–E**).

#### 3.1.4 Leaf weight ratio

**Figure 5:**
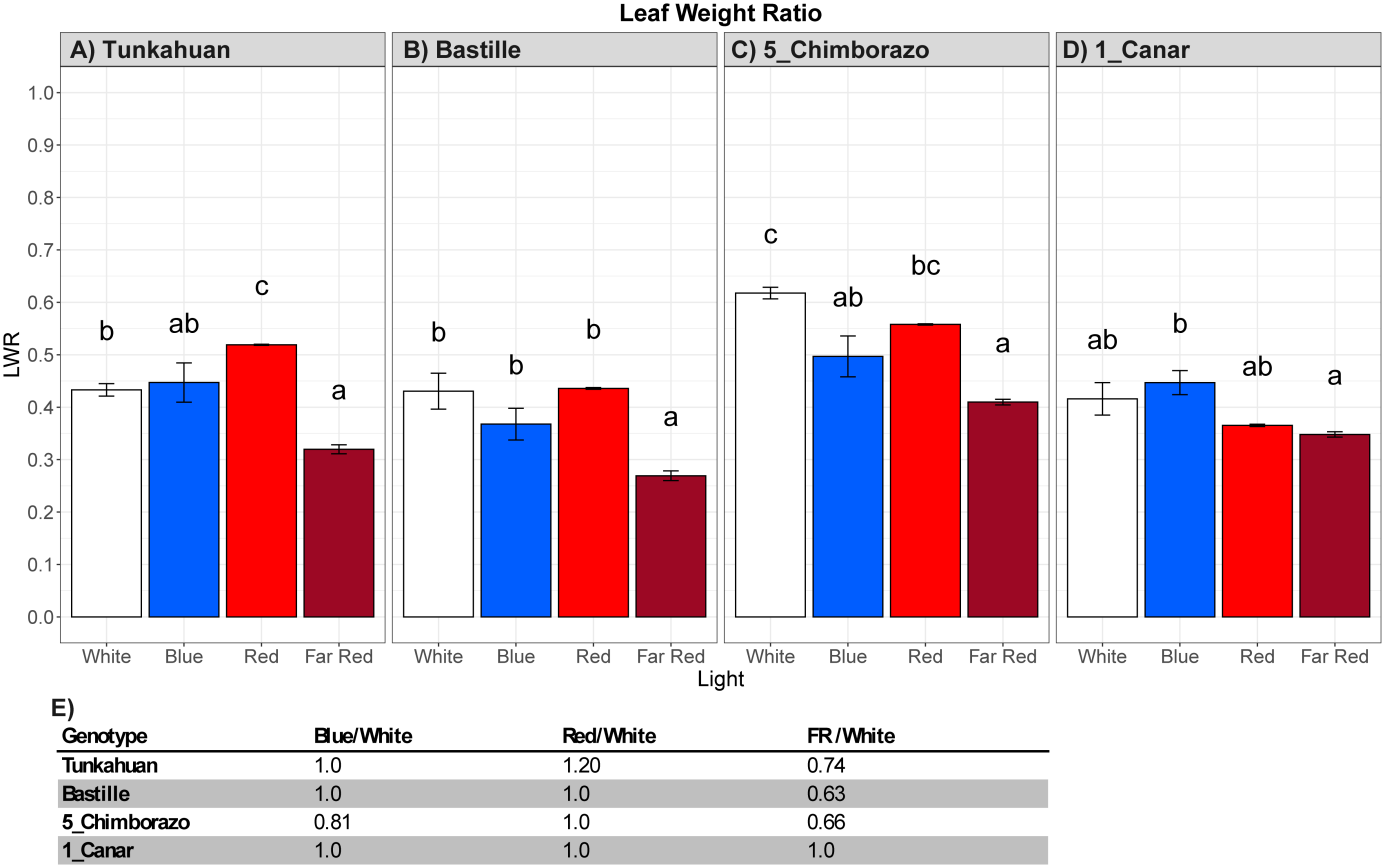
Leaf weight ratio (LWR) per genotype under four light treatments. **(A)** Tunkahuan, **(B)** Bastille, **(C)** 5_Chimborazo, **(D)** 1_Cañar. **(E)** Ratios of LWR under blue, red or far-red versus white control conditions; when differences between light treatments and white control were not significant for each genotype then ratios are equal to 1.0; when differences were statistically different the calculated ratios are shown. Different letters indicate significantly different values after ANOVA and Tukey tests. (p values: Tunkahuan 0.012, Bastille 0.001, 5_Chimborazo 0.008 and 1_Cañar 0.023).

Under white light, Leaf Weight Ratio (LWR, g leaves/g total biomass– DM) was highest in 5_Chimborazo (0.62) and similar among the other three genotypes (0.42) (**Supplementary Figure 3D**). Far-red supplementation significantly reduced LWR compared to the white control in three of the four genotypes. Reductions were of 26%, 37%, and 34% in Tunkahuan, Bastille, and 5_Chimborazo, respectively (**Figure 5E**). Under supplemental blue light, 5_Chimborazo exhibited a significant 19% reduction in LWR relative to white light (**Figure 5C, E**), while the other genotypes were unaffected. Under supplemental red light, the only significant difference compared to the control occurred in Tunkahuan, which showed a 20% increase in LWR (**Figure 5A, E**).

#### 3.1.5 Stem weight ratio

**Figure 6:**
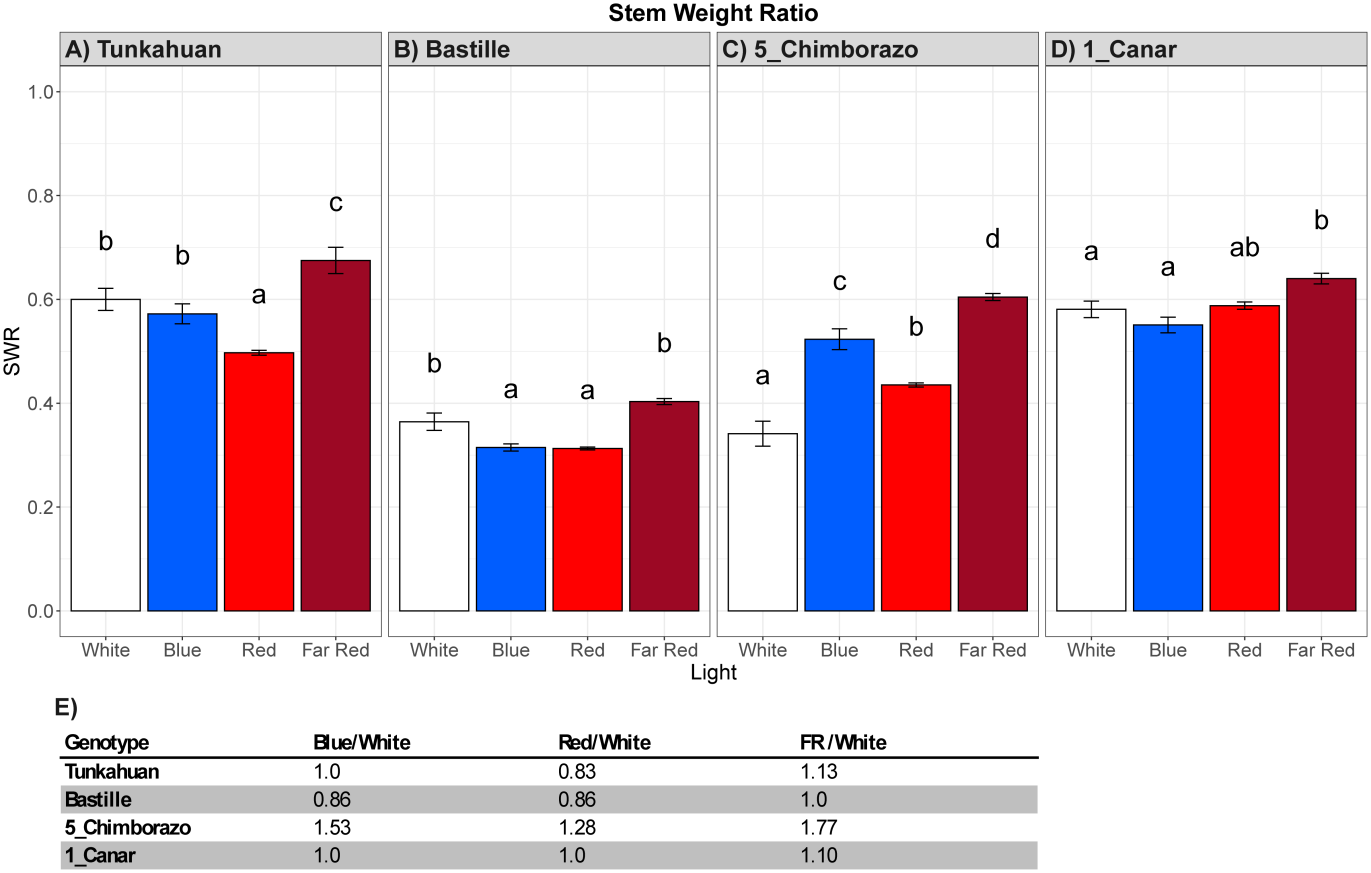
Stem weight ratio (SWR) per genotype under four light treatments. **(A)** Tunkahuan, **(B)** Bastille, **(C)** 5_Chimborazo, **(D)** 1_Cañar. **(E)** Ratios of SWR under blue, red or far-red versus white control; when differences between light treatments and white control were not significant for each genotype then ratios are equal to 1.0; when differences were statistically different the calculated ratios are shown. Different letters indicate significantly different values after ANOVA and Tukey tests. (p values: Tunkahuan <0.001, Bastille 0.001, 5_Chimborazo <0.001 and 1_Cañar <0.014).

Under white light, Stem Weight Ratio (SWR, g stem/g total biomass– DM) was higher in 1_Cañar (0.58) and Tunkahuan (0.60), and lower in 5_Chimborazo (0.34) and Bastille (0.36), **Supplementary Figure 3E**. Far-red light supplementation led to an increase in SWR in three genotypes. SWR increased by 13%, 77%, and 10% in Tunkahuan, 5_Chimborazo, and 1_Cañar, respectively, compared to the white control, while Bastille remained unaffected (**Figure 6E**). Supplemental red and blue light elicited contrasting responses among the four genotypes (**Figure 6**). Red light supplementation reduced SWR by 17% and 14% in Tunkahuan and Bastille, respectively, increased SWR by 28% in 5_Chimborazo, and had no effect on 1_Cañar relative to white light (**Figure 6E**). Under supplemental blue light, Bastille exhibited a 14% reduction in SWR, whereas 5_Chimborazo showed a 53% increase. The other two genotypes behaved as under white light (**Figure 6**).

#### 3.1.6 Relative growth rate

**Figure 7:**
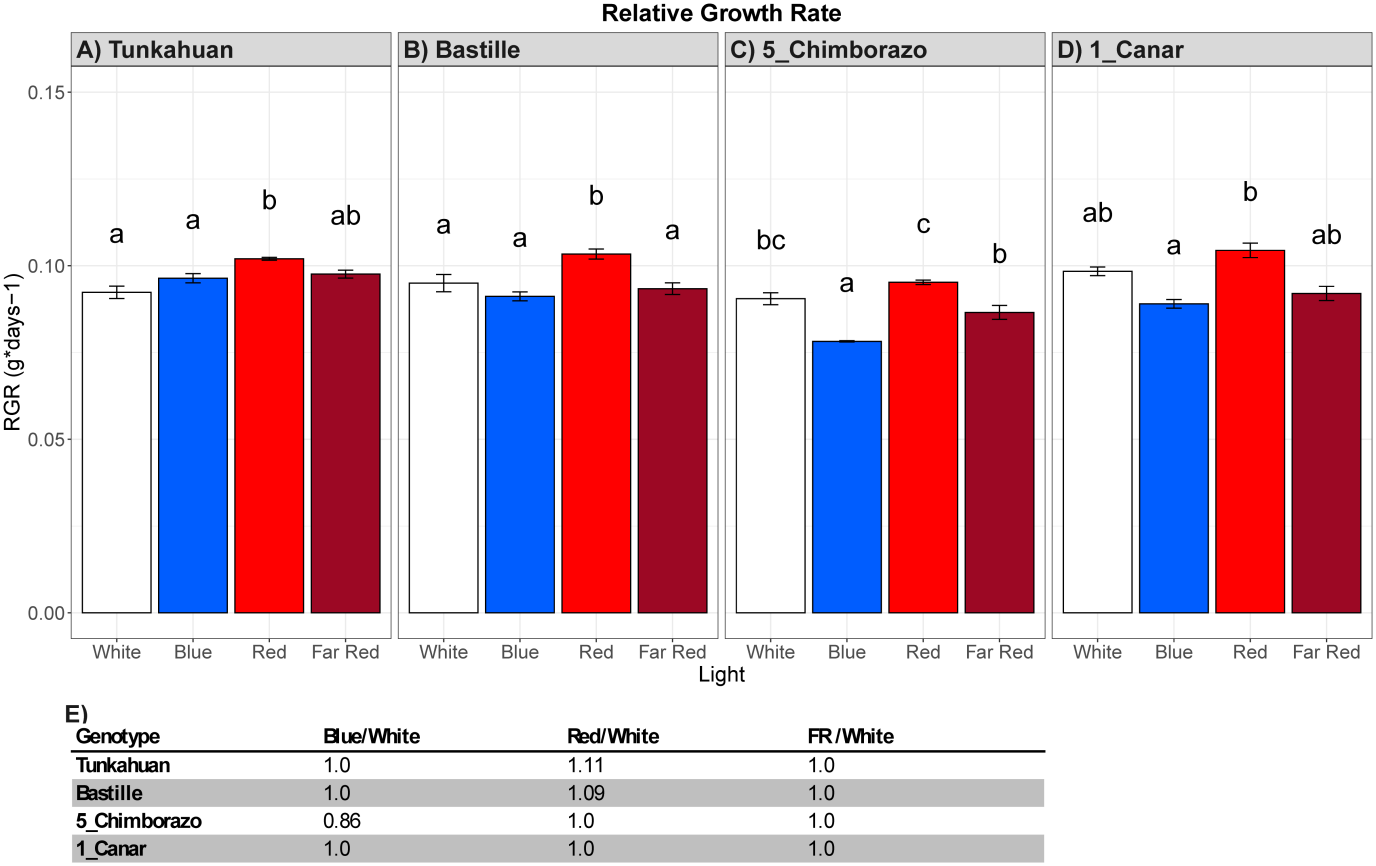
Relative growth rate (RGR) per genotype under four light treatments. **(A)** Tunkahuan, **(B)** Bastille, **(C)** 5_Chimborazo, **(D)** 1_Cañar. **(E)** Ratios of RGR under blue, red or far-red versus white control; when differences between light treatments and white control were not significant for each genotype then ratios are equal to 1.0; when differences were statistically different the calculated ratios are shown. Different letters indicate significantly different values after ANOVA and Tukey tests. (p values: Tunkahuan 0.001, Bastille 0.002, 5_Chimborazo 0.006 and 1_Cañar <0.001).

The four genotypes exhibited similar Relative Growth Rate (RGR, g days^-1^) (between 0.09 g days^-1^ and 0.10 g days^-1^) under white light (**Supplementary Figure 3F**). Supplemental light treatments resulted in minimal differential responses across genotypes, and their effects were milder compared to the other plant traits described above (**Figure 7**; **Supplementary Figure 3F**). 5_Chimborazo exhibited a statistically significant 14% reduction in RGR under blue light compared to the control (**Figure 7C, E**). Under red light, Tunkahuan and Bastille exhibited increases in RGR of 11% and 9%, respectively (**Figure 7A, B, E**).

#### 3.1.7 Net assimilation rate

**Figure 8:**
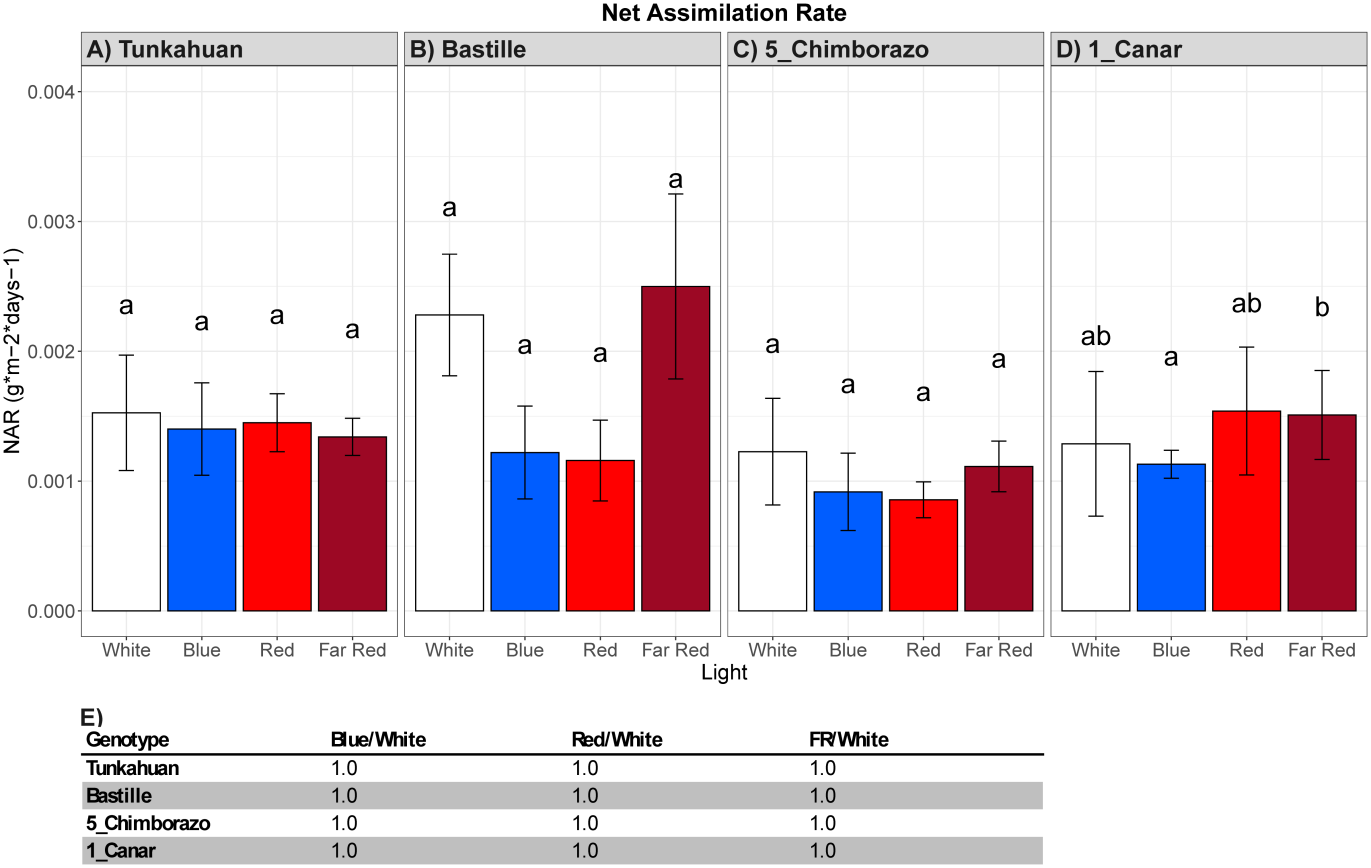
Net assimilation rate (NAR) per genotype under four light treatments. **(A)** Tunkahuan, **(B)** Bastille, **(C)** 5_Chimborazo, **(D)** 1_Cañar. **(E)** Ratios of NAR under blue, red or far-red versus white control conditions; when differences between light treatments and white control were not significant for each genotype then ratios are equal to 1.0; when differences were statistically different the calculated ratios are shown. Different letters indicate significantly different values after ANOVA and Tukey tests. (p values: Tunkahuan 0.979, Bastille 0.160, 5_Chimborazo 0.773 and 1_Cañar 0.887).

Net Assimilation Rate (NAR, g m^-^² day^-^¹) differed among the four genotypes under white light. Bastille exhibited the highest NAR (0.0022 g m^-^² day^-^¹), 80% higher than that of 5_Chimborazo, which had the lowest NAR (0.0012 g m^-^² day^-^¹). NAR of Tunkahuan was 0.0015 and of 1_Cañar 0.0013 g m^-^² day^-^¹ (**Supplementary Figure 3G**). None of the three supplemental light conditions caused statistically significant changes in NAR for any of the four genotypes compared to the white control (**Figure 8E**).

### 3.2 Impact of salinity on physiological responses to light

**Figure 9:**
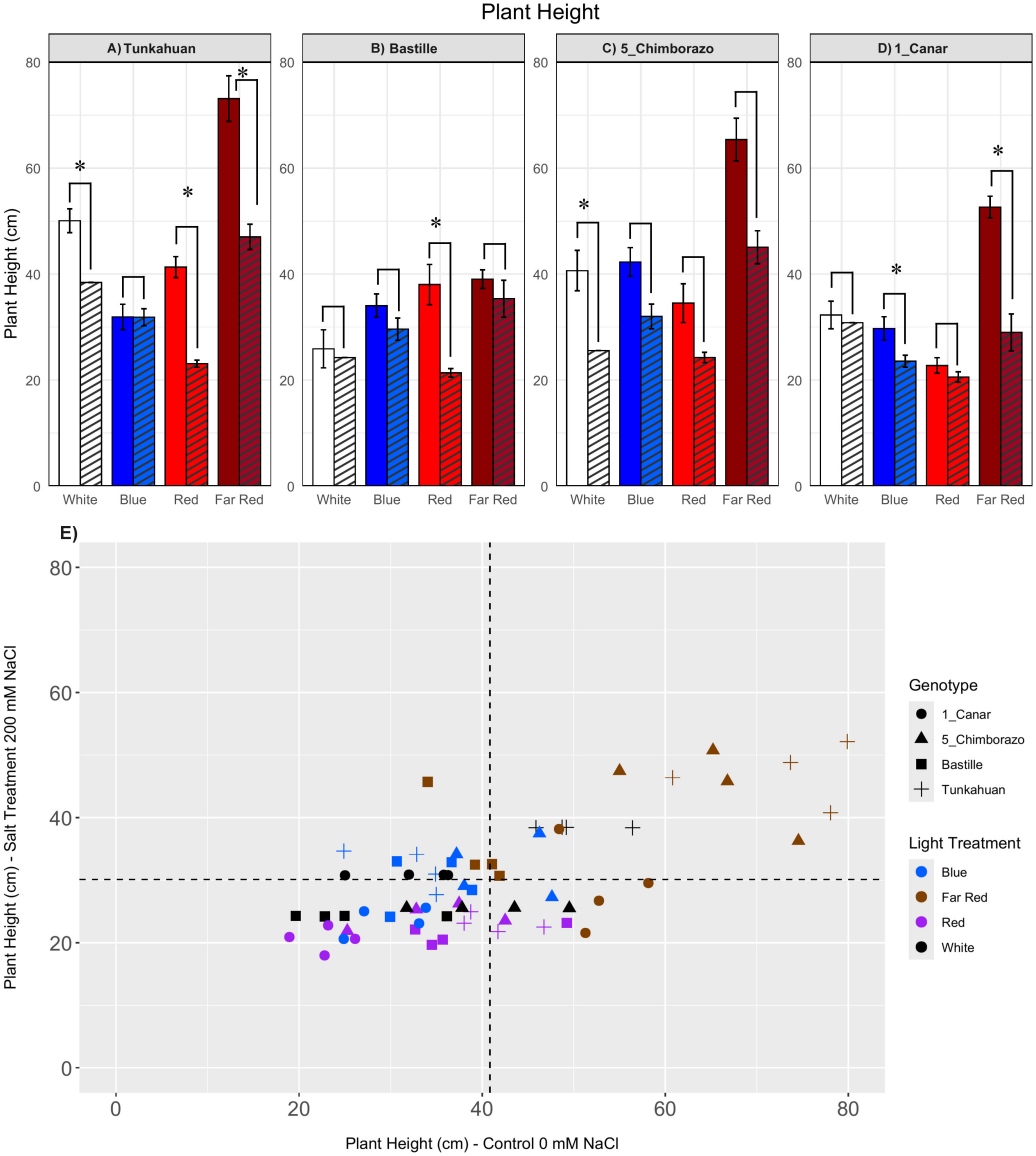
Effect of salt on plant height per genotype under four light treatments. **(A)** Tunkahuan, **(B)** Bastille, **(C)** 5_Chimborazo, **(D)** 1_Cañar under the absence and presence of salt and under white and supplementary blue, red, and far-red light. Values obtained under control and salinity conditions were compared using Student’s t-test or Kruskal-Wallis test, depending on data normality. Asterisks indicate statistically significant differences between control and treatment. **(E)** Two-dimensional scatterplot. Each point represents plant height under a specific light treatment (shown by color) and by genotype (shown by symbol). The x-axis represents absence of salt, the y-axis presence of salt. Dashed vertical and horizontal lines indicate the overall mean for control and salt conditions, respectively. The dashed lines the plot into four quadrants: (1) upper right—plants performing above average in both conditions, that is, under the absence and presence of salt; (2) upper left—plants performing better under salt than under no salt; (3) lower right—plants performing better under no salt than under salt; and (4) lower left—plants performing below average in both the absence and the presence of salt.

**Figure 10:**
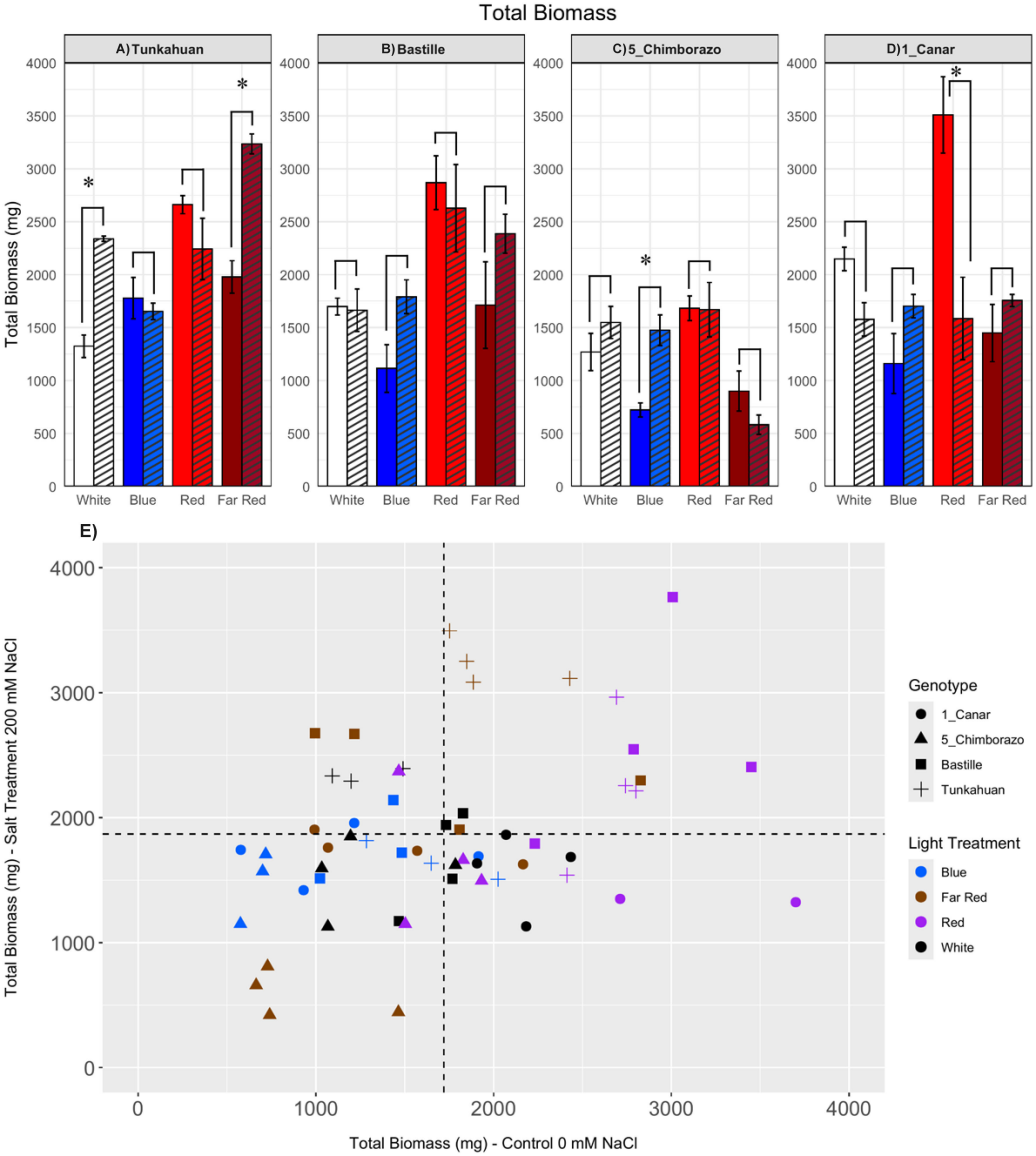
Effect of salt on total biomass per genotype under four light treatments. **(A)** Tunkahuan, **(B)** Bastille, **(C)** 5_Chimborazo, **(D)** 1_Cañar under the absence and presence of salt and under white and supplementary blue, red, and far-red light. Values obtained under control and salinity conditions were compared using Student’s t-test or Kruskal-Wallis test, depending on data normality. Asterisks indicate statistically significant differences between control and treatment. **(E)** Two-dimensional scatterplot. Each point represents total biomass under a specific light treatment (shown by color) and by genotype (shown by symbol). The x-axis represents absence of salt, the y-axis presence of salt. Dashed vertical and horizontal lines indicate the overall mean for control and salt conditions, respectively. The dashed lines the plot into four quadrants: (1) upper right—plants performing above average in both conditions, that is, under the absence and presence of salt; (2) upper left—plants performing better under salt than under no salt; (3) lower right—plants performing better under no salt than under salt; and (4) lower left—plants performing below average in both the absence and the presence of salt.

To investigate responses to light quality under salinity, the four genotypes were subjected to the same four light treatments in the presence of a moderate salt concentration (200 mM NaCl). **Supplementary Figure 4** displays plant height (panel S4A), total biomass (S4B), SLA (S4C), LWR (S4D), SWR (S4E), RGR (S4F), and NAR (S4G). Similarly to what was seen under the absence of salt, supplemental light treatments modified quinoa traits in a genotype-specific manner compared to white light (**Supplementary Figure 5**-**S11**; **Table 1**). To better visualize the effects of salinity on plant growth across the four genotypes and the light conditions tested, we generated two-dimensional scatterplots for each of the plant traits measured, comparing control and salt treatments (**Figures 9**, **10**; **Supplementary Figures 12–S15**). These visualizations highlight and summarize differential responses to salinity across the four genotypes and illustrate how these responses vary under different light conditions for each trait.

**Table 1:** Ratios of seven quinoa traits under supplemental blue, red or far-red light versus white control conditions under the presence of salt (200 mM NaCl).

Traits	Genotype	Blue/White	Red/White	FR/White
Plant Height	Tunkahuan	0.83	0.6	1.22
	Bastille	1.0	1.0	1.46
	5_Chimborazo	1.0	1.0	1.76
	1_Canar	1.0	0.67	1.0
Total Biomass	Tunkahuan	1.0	1.0	1.0
	Bastille	1.0	1.0	1.0
	5_Chimborazo	1.0	1.0	0.38
	1_Canar	1.0	1.0	1.0
SLA	Tunkahuan	1.0	1.0	1.0
	Bastille	1.0	0.5	1.0
	5_Chimborazo	1.0	1.0	3.41
	1_Canar	0.4	1.0	1.0
LWR	Tunkahuan	1.15	1.0	0.79
	Bastille	1.12	1.12	0.69
	5_Chimborazo	1.0	1.0	0.83
	1_Canar	1.16	1.0	0.77
SWR	Tunkahuan	0.83	1.0	1.22
	Bastille	1.0	1.0	1.0
	5_Chimborazo	1.0	1.0	1.27
	1_Canar	0.81	1.0	1.26
RGR	Tunkahuan	0.93	1.0	1.0
	Bastille	1.0	1.0	1.0
	5_Chimborazo	1.0	1.0	0.82
	1_Canar	1.0	1.0	1.0
NAR	Tunkahuan	1.0	1.0	1.0
	Bastille	1.0	1.0	1.0
	5_Chimborazo	1.0	1.0	1.0
	1_Canar	1.0	1.0	1.0

When differences between light treatments for each genotype were not significant then ratios are equal to 1.0. When differences were statistically different the calculated ratios are shown.

#### 3.2.1 Plant height

When comparing plant height under white light between control and salt-treated plants, Tunkahuan and 5_Chimborazo showed reductions of 23% and 37%, respectively, when salt was added (**Figure 9A, C**), whereas Bastille and 1_Cañar were unaffected (**Figure 9B, D**). Under supplemental blue light, Tunkahuan, Bastille and 5_Chimborazo showed similar plant height with and without salt (**Figures 9A–C**), while 1_Cañar exhibited a 21% reduction in plant height in the presence of salt (**Figure 9D**). Under supplemental red light, the presence of salt reduced plant height in Tunkahuan and Bastille by 44% each (**Figure 9A, B**, but had no effect on 5_Chimborazo and 1_Cañar (**Figure 9C, D**). The presence of salt similarly significantly decreased plant height in Tunkahuan and 1_Cañar under far-red light by 36% and 45%, respectively (**Figure 9A, D**), but did not affect Bastille and 5_Chimborazo (**Figure 9B, C**). The two-dimensional scatterplot (**Figure 9E**) shows that far-red light increased plant height in both the absence and presence of salt for 5_Chimborazo and Tunkahuan. In 1_Cañar, far-red light increased plant height only under non-saline conditions. Plant height values for all four genotypes under blue and white light treatments were distributed near the salt/no salt means, while red light generally resulted in reduced plant height across the genotypes.

#### 3.2.2 Total biomass

Total biomass did not differ significantly between salt-treated and control plants across the four genotypes and light conditions (**Figures 10A–D**). However, some genotype-specific responses were observed. Under white and far-red light, salinity increased total biomass in Tunkahuan by 76% and 64%, respectively. In contrast, supplemental blue light increased biomass in 5_Chimborazo by 104%, while red light reduced biomass in 1_Cañar by 55%. Compared to plant height, changes in total biomass due to salinity were less pronounced. Red light caused an effect opposite to that observed for plant height, resulting in increased biomass regardless of salt treatment (**Figure 10E**). Blue light effects on biomass mirrored those seen for plant height.

#### 3.2.3 Specific leaf area and leaf weight ratio

The growth parameters SLA and LWR were analyzed together, as they reflect leaf structure and biomass allocation, respectively. Supplemental red and far-red light caused contrasting effects (**Supplementary Figures 12E**, **S13E**). For all four genotypes, the observed trend was that under far-red light, plants had fewer thinner leaves (higher SLA, lower LWR), while red light promoted higher leaf mass and thicker leaves (lower SLA, higher LWR). Across all light treatments, all four genotypes produced greater leaf biomass under salt conditions compared to the control (absence of salt) (**Supplementary Figure 13**). However, leaf characteristics varied by genotype and light type. For instance, under far-red light, specific leaf area (SLA) was lower in salt-treated plants, indicating thicker leaves, whereas under red light, salt-treated plants had thinner leaves compared to control conditions (**Supplementary Figure 12**).

#### 3.2.4 Net assimilation rate

Net Assimilation Rate (NAR), an indicator of productivity and photosynthetic efficiency, showed distinct responses across light treatments and genotypes (**Supplementary Figures 14A–D**). NAR was generally lower under the presence of salt compared to the absence of salt (**Supplementary Figures 14A–D**). Under white light, salt reduced NAR only in Bastille (45% reduction), whereas under far-red light, salt-treated Tunkahuan and Bastille plants exhibited reductions of 47% and 34%, respectively. In contrast, supplemental red light caused NAR to increase in the presence of salt, while far-red light decreased it (**Supplementary Figure 14E**). As observed for other plant traits, NAR under blue light closely resembled values measured under white light.

#### 3.2.5 Relative growth rate

Overall, RGR exhibited distinct responses across light treatments and genotypes (**Supplementary Figures 15A–D**). Under white light, Tunkahuan showed a 9% increase in RGR when comparing salt to control conditions. Supplemental blue light increased RGR in 5_Chimborazo and 1_Cañar by 19% and 8%, respectively. In contrast, supplemental far-red light increased RGR in Tunkahuan by 8% but reduced RGR in 5_Chimborazo by 9%.

## 4 Discussion

Photobiology supports the optimization of cultivation practices in both controlled environments and open-field agriculture. It can aid in the development of improved genotypes through two key approaches: breeding plants with enhanced responsiveness to light and environmental inputs to achieve higher yield and quality, and investigating the underlying physiological and metabolic mechanisms that drive these improvements (Folta, 2024). When manipulating light parameters to improve crop growth and quality, researchers and growers are often faced with the problem that different light environments affect plants with species- and genotype-dependent effects (Cervantes et al., 2019; Kong and Zheng, 2019; Tsuruyama and Shibuya, 2023). Such effects hinder the translation of experimental results into broadly applicable and predictable applications. In the current work, we assessed the impact of four light conditions on quinoa early-stage, vegetative growth by analyzing seven growth components: plant height, total biomass, Specific Leaf Area (SLA), Leaf Weight Ratio (LWR), Specific Weight Ratio (SWR), Relative Growth Rate (RGR), and Net Assimilation Rate (NAR). The four landraces tested may have different inductive photoperiods and have very different life cycle durations, as described in Section 2.1. Therefore, for this study the focus was placed on vegetative growth rather than on flowering and yield-related parameters. The findings reinforce the complexity of effects of light signaling quality on quinoa growth, development and physiology, as four genotypes (Tunkahuan, Bastille, 5_Chimborazo and 1_Cañar) exhibited distinct responses when subjected to different light treatments. Notwithstanding, the observed differential responses, particularly under saline conditions, can be leveraged to dissect the diverse metabolic pathways governing growth, providing crucial insights for designing quinoa cultivars with enhanced resilience to adverse conditions.

The four light conditions used a white light 5700 K channel as baseline to analyze effects of added blue, red, and far-red supplemental light (**Supplementary Figure 1A**). Total light intensity (μmol m^2^ s^-1^) cannot be used as a factor to compare light conditions, but rather light quality, or spectrum, through the relative contributions of blue, red, and far-red light (**Supplementary Figure 1B**). The white light 5700 K channel had a contribution of B:R:FR of 46:41:13. Supplemental blue light generated the spectrum of 64:27:9, supplemental red light 31:60:9, and supplemental far-red light 31:27:42. Using the 46:41:13 spectrum as baseline was determined by the commercial solutions available for the current study. Although green light ratios also influence plant growth and performance, this region of the spectrum was not considered for this manuscript (Wang and Folta, 2013; Smith et al., 2017; Kusuma et al., 2021). Similarly, quinoa’s ability to grow in the high Andes may be linked to unique responses to high UV light, which should be investigated in future studies (Parra et al., 2019).

Quinoa is typically grown, and has evolved, in open-field conditions under full sunlight, and full solar spectrum. Light intensity fluctuates during the day in the field and is particularly high in the Andes, reaching over 3,000 μmol m^-2^ s^-1^, which is over 10 times higher than the light intensity tested in the current trial (Parra et al., 2019). The tested genotypes have different geographical origin and are adapted to different daylength and temperature regimes. The tested conditions did not intend to replicate ecologically relevant scenarios but rather creating controlled environments and management practices to allow us investigating the role of light quality in quinoa development and a potential interaction with responses to salinity.

Under the absence of salt, plant height increased in all four genotypes under supplemental far-red light compared to the white light control. Supplemental far-red light resulted in a ratio R:FR of 0.64, which mimics moderate shade and vegetation proximity, suggesting quinoa displayed shade-avoidance behavior (Franklin, 2008; Martinez-Garcia and Rodriguez-Concepcion, 2023). Such observation is not consistent with other reports in the literature. A previous study stated that quinoa plants decreased plant height under high plant density, which caused light competition (González et al., 2022), whereas other authors did not see impacts of plant density on plant height (Asher et al., 2022). Such reports did not specify ratios of R:FR experienced by plants under different densities and used other quinoa genotypes. Other factors that may have confounded results may have been looking at different developmental stages, and the usage of different light spectra, with an emphasis on green light contribution, different light intensity, and other environmental setpoints known to affect how plants respond to full light and shade conditions (Shibuya et al., 2019; Kusuma et al., 2021; Martinez-Garcia and Rodriguez-Concepcion, 2023). In the present study, all four quinoa genotypes displayed shade-intolerant behavior, consistent with the breeding history of quinoa in open-field conditions in the high Andes (Jacobsen, 2003; Gomez-Pando et al., 2019; Angeli et al., 2020). To further understand the basis of quinoa’s response to shade, it would be advisable to test lower R:FR ratios to simulate deeper shade, as well as other combinations of blue and green light, and reduced light intensity (Kusuma et al., 2021; Martinez-Garcia and Rodriguez-Concepcion, 2023). Additional traits beyond stem elongation, part of the shade avoidance syndrome (SAS), could be evaluated, such as inhibition of leaf expansion, adjustment of photosynthetic metabolism, and induction of flowering, all of which have been described in Arabidopsis, tomato, and other shade-intolerant species.

Total biomass was overall affected by supplemental red light, but not by blue light, under the absence of salt when compared to white light. It increased in three genotypes (Tunkahuan, Bastille, 1_Cañar) but was unaffected in 5_Chimborazo. Red light might have improved biomass though increased photosynthetic rates coupled with phytochrome activity (Yang et al., 2016). Extensive research has tested different combinations and contributions of blue and red light, and other wavelengths, using sole source bandwidths or broad spectra, on several plant species (Moradi et al., 2021; Yavari et al., 2021; Mansoori et al., 2023). Photosynthesis is promoted by several regions of the spectra, including blue, green, red and far-red photons (Kochetova et al., 2022). Red photons are more efficient and usually preferred in controlled environment agriculture (Kusuma et al., 2020, 2023). But red light is usually not enough to support proper growth, resulting in the so-called red-light syndrome, characterized by a dysfunction of the photosynthetic machinery, low carbon assimilation and unresponsive stomatal conductance (Hogewoning et al., 2010; Miao et al., 2019). The right proportion of blue light may be hard to identify. Excessive blue light results in lower efficacy and may hinder plant growth (Kusuma et al., 2020). In cucumber 50% blue light has been described as the maximum contribution to have in the spectrum, as higher percentages decrease photosynthetic assimilation (Hogewoning et al., 2010). Blue light at 40% has been pointed as the best light environment for the edible halophyte *Mesembryanthemum crystallinum* in terms of photosynthetic performance and leaf area and biomass accumulation (Zhang et al., 2021). Saffron (*Crocus sativus*), in opposite, shows enhanced photosynthetic performance, flower production and higher stigma yield with blue light contributions close to 100% (Moradi et al., 2021). The current work tested four B:R ratios (1.1, 2.4, 0.5, 1.2), as shown in **Supplementary Figure 1**, panel b. Among these four light treatments, biomass was highest under B:R of 0.5, suggesting 0.5 as a good B:R baseline for quinoa when targeting improved biomass.

While overall effects of light quality were clear when assessing plant height and biomass, the other plant traits analyzed showed fewer clear patterns in response to light quality and displayed strong genotype-specific effects when salt was not present. It is possible that the long photoperiod used may have affected responses to light quality, considering the genotypes tested have different geographical origin. Bastille is adapted to the northern European summer climate and long photoperiod (16h light/8h dark) (De Bock et al., 2021), whereas Tunkahuan and the Ecuadorian landraces 5_Chimborazo and 1_Cañar are suited to Andean high-altitude climate conditions and short photoperiod (12h light/12h dark) (Villacrés et al., 2022). Bastille is an early-maturing, long-day genotype that progresses through its developmental stages more rapidly, which results in a faster vegetative growth rate, shorter grain-filling period, and quicker senescence. In contrast, Tunkahuan and the Andean landraces (1_Cañar and 5_Chimborazo) are late-flowering, short-day–adapted materials typical of the Inter-Andean Valley ecotype and have substantially longer vegetative and grain-filling phases. Under white light and in the absence of salt, Bastille accumulated more biomass despite its shorter stature. This pattern is consistent with inherent characteristics of the variety: as an early-maturing genotype, Bastille progresses more rapidly through its developmental stages, and its reduced plant height reflects breeding for European mechanized production systems. These intrinsic traits likely explain why Bastille showed a stronger reduction in NAR under white light with salt. Because it develops faster, Bastille is probably at a more advanced physiological stage when stress occurs, making it more susceptible than the slower-developing Andean genotypes. Although such phenological differences help interpret part of the variation observed between Bastille and the late-flowering Andean materials, they do not account for the broader trends in the dataset. Across light-quality treatments, the Andean genotypes exhibited distinct responses to changes in spectral composition, both under salt and no-salt conditions, indicating that their sensitivity to light quality is not uniform. Taken together, these results show that light quality elicits strongly genotype-specific responses, and that while phenology contributes to some contrasts—particularly for Bastille—the predominant driver of variation is the interaction between genotype and light environment. Future research should explore a range of light quality regimes under both long- and short-day conditions, reflecting the diversity of light environments where quinoa is grown.

Light is known to affect plant responses to salt, but such relationship has not been investigated in quinoa (Qiu et al., 2023; Liang et al., 2024). 200 mM NaCl, a moderate salt level, was chosen to trigger salt response mechanisms, such as increased mesophyll thickness and Na^+^ accumulation, but to not affect quinoa growth to a large extent given the crop capacity to grow under the presence of salt (Adolf et al., 2013; Böhm et al., 2018; Manaa et al., 2019; Cai and Gao, 2020; López-Marqués et al., 2020; Hussin et al., 2023; Terletskaya et al., 2023). The presence of salt under white light led to observable physiological changes in the genotypes tested without causing significant, damaging stress. Relative growth rate (RGR), which is a simplified model of growth, showed limited variation under the presence and absence of salt. On average, a vegetative quinoa plant grows at a rate of 0.1 g fresh weight/day (i.e., 10% daily increase) (Jaramillo Roman, 2021) and 200 mM did not affect this rate. Plant height was reduced in Tunkahuan and 5_Chimborazo under the presence of salt, and unaffected in the other two genotypes, while total biomass increased in Tunkahuan and did not change in the other three genotypes. Such observations may be linked to changes in plant architecture, such as thicker leaves and/or stems as a result of salt accumulation in anatomical structures (Negrão et al., 2017; Terletskaya et al., 2023).

The three supplemental light conditions caused in some instances responses to salt to shift with high genotype-dependent effects, and without clear overall trends. 1_Cañar showed the same height under supplemental far-red and white light, suggesting the shade avoidance behavior may have been abolished under the presence of salt – something that did not occur in the other three genotypes. Understanding the molecular pathways that make these genotypes differ in this specific behavior may allow pinpointing targets for breeding linked to shade avoidance, yield, and salt tolerance. Plants had fewer and thinner leaves (lower LWR, higher SLA) under the presence of salt and supplemental far-red light but higher leaf mass and thicker leaves (higher LWR, lower SLA) under red light. These changes may reflect differences in photosynthetic efficiency and growth, as thicker leaves often indicate enhanced metabolic activity due to more photosynthetically active tissue (Momayyezi et al., 2022; Zhang Y. et al., 2022; Ding et al., 2025). The observed shift in leaf morphology under salinity and supplemental far-red light highlights a potential protective effect of far-red light on quinoa growth when salt is present. Far-red light has been shown to interact with salinity responses in several crops, such as tomato and cucumber, through phytochrome B, and may be used as a drought stress mitigator in horticultural crops (González et al., 2012; Liu et al., 2012; Cao et al., 2018; Qiu et al., 2023; Shibuya et al., 2024). In future studies, it would be valuable to compare the four genotypes at both molecular and physiological levels to investigate potential differences in phytochrome activity, and how these may relate to osmotic adjustment and photosynthetic regulation under the absence and presence of salt.

The positive effect caused by red light on total biomass under the absence of salt was not seen in the presence of salt. Red light may not be as effective on biomass when salt is present due to expected reduced photosynthetic rates and/or due to potential impairment of phytochrome signaling (Eisa et al., 2012; Ma et al., 2023). There are reports describing interactions between red light and salt stress responses, with the HY5-COP1 pathway being a central player (Indorf et al., 2007; Carvalho et al., 2013). But when plants were exposed to supplemental blue light, total biomass was comparable in the absence and presence of salt consistently across all four genotypes. This suggests that blue light signaling may be able to counteract potential impairment of red light-driven photosynthesis and promotion of biomass under the presence of salt (Peng et al., 2016; Malekzadeh Shamsabad et al., 2022). The HY5-COP1 light signaling pathway, which also acts downstream of cryptochrome (Bhatnagar et al., 2020), may be an interesting path to explore when looking in the future at photoreceptor activity and molecular players in response to light and salinity.Salinity reduces photosynthetic rates in quinoa, and other crops, and growth rates due to RubisCO impaired carboxylation and/or content, reduced RuBP regeneration, reduced photosystem II (PSII) performance, and low photosynthate supply (Downton, 1977; Eisa et al., 2012; He et al., 2016; Becker et al., 2017; Killi and Haworth, 2017; Manaa et al., 2019; Esmaeilizadeh et al., 2021; Pan et al., 2021; Hussin et al., 2023). In soybean, a moderately salt-tolerant crop, higher salt tolerant lines maintain higher photosynthetic and stomatal conductance rates under salt, which are coupled to yield-related traits (He et al., 2016). Blue light regulates chloroplast structure, photosynthesis and stomata aperture through different photoreceptors and signaling pathways (Inoue and Kinoshita, 2017; Miao et al., 2019; Moosavi-Nezhad et al., 2021; Moradi et al., 2021; Zhang L. X. et al., 2022). In Arabidopsis the sigma factor SIG5, which regulates expression of photosynthesis genes in chloroplasts, protects chloroplasts under various stress conditions, including salinity, through enhancement of PSII reaction center repair (Nagashima et al., 2004; Tsunoyama et al., 2004). *SIG5* expression is induced by blue light, but not red light, and is necessary for the activation of BLRP, a blue light-responsive promoter, acting downstream of cryptochrome to regulate gene expression in the chloroplast (Tsunoyama et al., 2004).

Salt stress impacts other physiological traits besides photosynthesis. It damages thylakoid membranes, which induces oxidative stress (Pospíšil, 2009; Killi and Haworth, 2017). Antioxidants protect plants against salinity in quinoa and other plants (Kan et al., 2012; Amjad et al., 2015; Xu et al., 2016). Blue light signaling has been linked to antioxidant protection in various crops, such as basil, lettuce, saffron, and vanilla (Ko et al., 2020; Moradi et al., 2021; Rusaczonek et al., 2021; Chutimanukul et al., 2022; Anum et al., 2024). Cryptochrome 2 mediates degradation of antioxidant betacyanins under blue light in seedlings of the halophyte *Suaeda salsa* (Chang-Quan and Tao, 2006). Betacyanins protect plants against abiotic stress and are present in quinoa, being linked to the diversity of grain color in the crop (Escribano et al., 2017). Betacyanins decrease in quinoa epidermal bladder cells under salt stress (Otterbach et al., 2021). Proline is an osmoprotectant that supports tolerance to salt stress in quinoa and other crops (Iqbal, 2017; Abdallah et al., 2020). Proline levels are regulated by light, and the HY5-COP1 pathway functions in this regulatory process (Bhatnagar et al., 2020). In Arabidopsis, blue and red, but not far-red light, activate HY5, which promotes salt induced-proline biosynthesis by upregulating *P5CS1*, involved in proline biosynthesis, and downregulating *PDH1*, involved in proline catabolism (Kovács et al., 2019). Light also regulates transcriptional memory of proline accumulation to repeated salt stresses through epigenetic modifications of *P5CS1*, a mechanism that allows plants to respond more efficiently to abiotic stress (Feng et al., 2016).

Future work should challenge quinoa to increasing salt concentrations and use a mix of more and less salt tolerant genotypes to push for more striking phenotypical differences. Traits related to photosynthetic efficiency, such as chlorophyll fluorescence, chloroplast structure, PSII performance, electron transport rate, stomata density, photosynthetic assimilation, and stomatal conductance can be analyzed under different light conditions (Manaa et al., 2019). Based on our initial observations, quinoa plants exposed to high salt stress may perform better under supplemental blue and/or far-red light than under red light. Knockout/silencing lines of phytochrome and cryptochrome in quinoa should be considered. Allelic diversity of light receptors, protein conformations, downstream pathways, particularly the HY5-COP1 pathway, and transcriptional memory to repeated salt stress, should be investigated across quinoa genotype collections (Feng et al., 2016; Bhatnagar et al., 2020). Yield parameters, including grain-related traits, protein, saponin, starch and sugar contents, should be assessed in the future too, as well as root development. Similarly to what was observed in this study, quinoa has been reported to show contrasting root development in response to salinity among different genotypes (Nguyen et al., 2020; Comparini et al., 2024). Light affects root development and plasticity (van Gelderen et al., 2018). Such complex interactions might unravel unique mechanisms in quinoa.

Depending on specific plant traits of interest, the strategy described in this manuscript can be used to uncover genotype-specific signaling behaviors that regulate quinoa responses to light and salt. The knowledge might be useful to generate other food crops more resistant to salinity, as well as for ecological studies and conservation approaches (López-Hoffman et al., 2007). Quinoa breeding programs may benefit from generating different genotypes prone to be grown in different geographical locations for enhanced access to nutritious food, as light conditions (quality, intensity, duration) vary drastically with latitude. Usage of controlled environments may also facilitate speed breeding and faster obtention of targeted genotypes, particularly with the usage of sequential light recipes that target different crop developmental stages (Ghosh et al., 2018; Chiurugwi et al., 2019).

## Data Availability

The original contributions presented in the study are included in the article/**Supplementary Material**. Further inquiries can be directed to the corresponding author.
