# Supplementary material for: Genotype by light quality interaction on the growth and development of quinoa (Chenopodium quinoa) and the crop response to salinity
Source: Front Plant Sci. 2026 Jun 12;17:1725934. doi: 10.3389/fpls.2026.1725934 (PMC13303818; doi:10.3389/fpls.2026.1725934)
Supplement: Supplementary file 1 [file DataSheet1.docx]

***Supplementary Material***


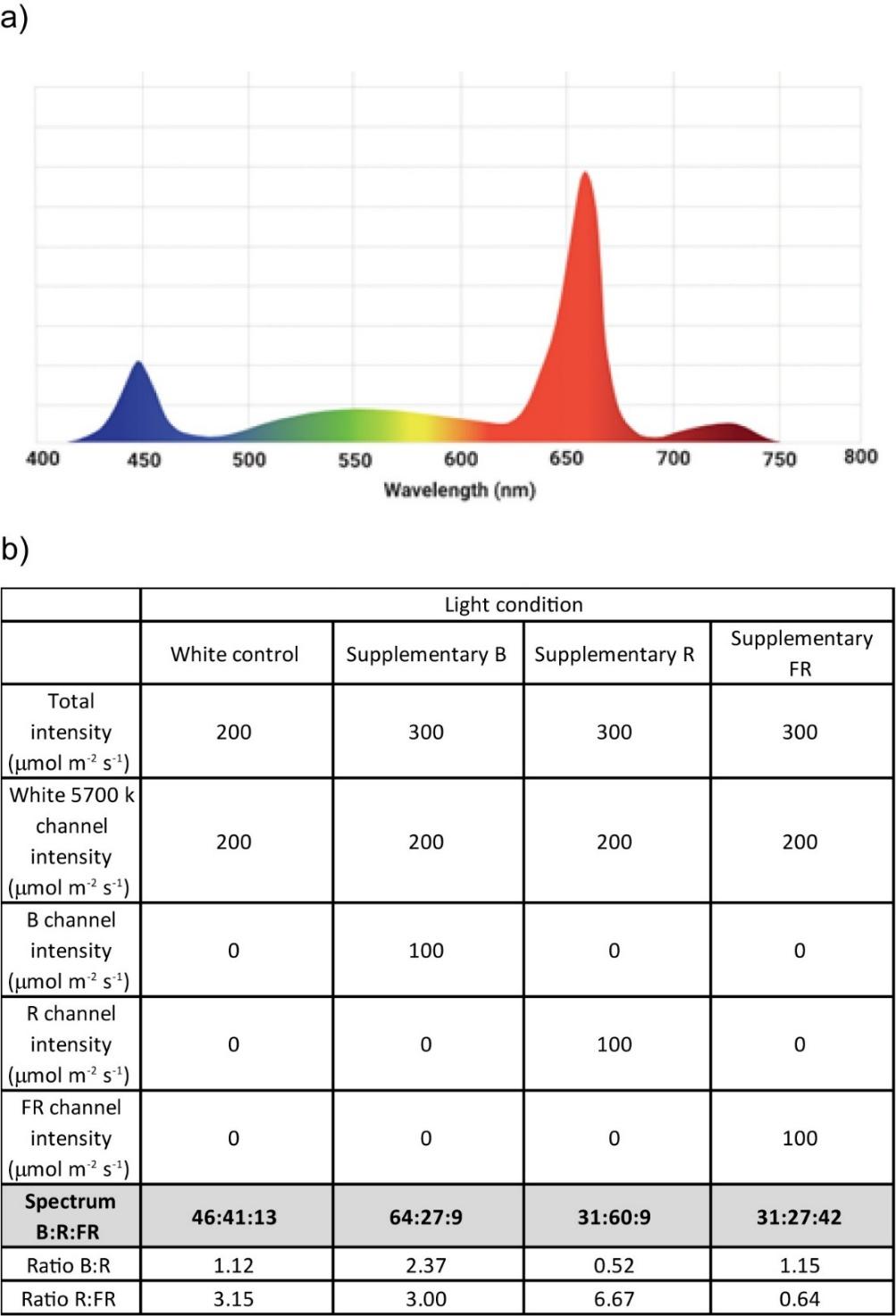


**Figure S1**. Light conditions used in the current work. (a) White light broad spectrum (5700 K) from Elixia lamps (Heliospectra). Independent channels peak at 450 nm (Blue), 660 nm (Red), and 735 nm (Far-red). Figure taken from https://heliospectra.com/led-grow-lights/elixia/. (b) Contributions of Blue (B) Red (R) and Far-red (FR) and ratios of Blue to Red (B:R) and Red to Far-red (R:FR) in the four light conditions tested.


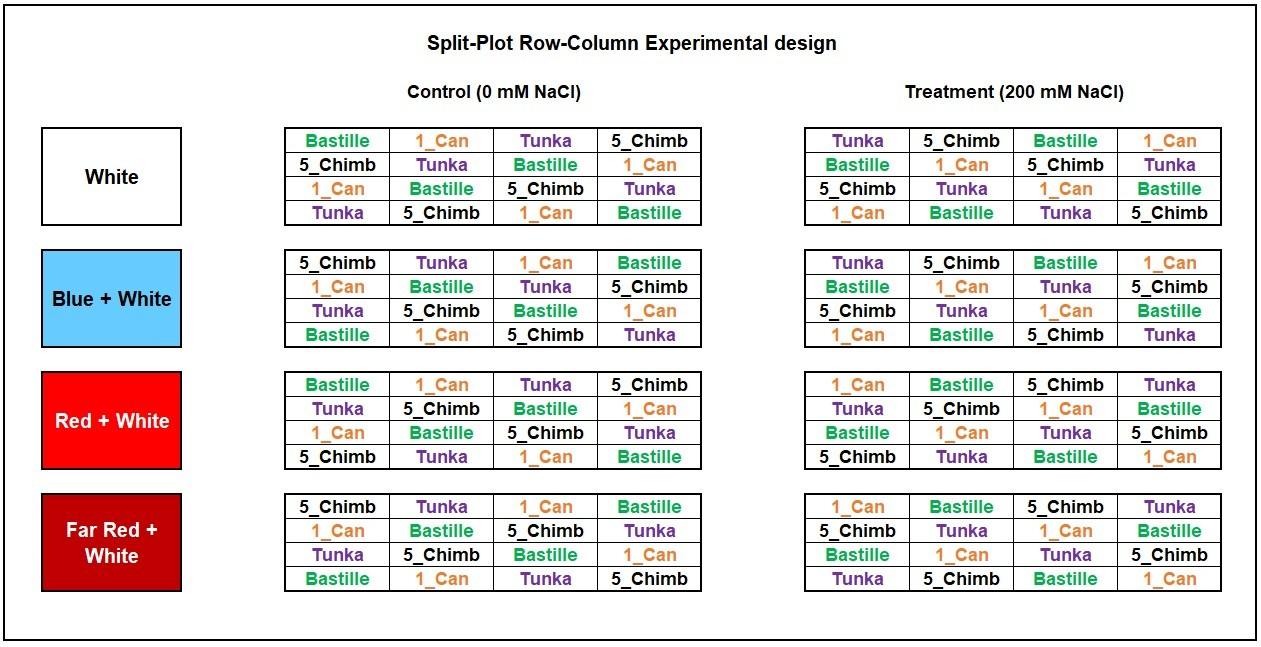


**Figure S2.** Split plot row-column design. Distribution of the Control (0mM NaCl) and Salt treatment (200mM NaCl) sections, containing the four quinoa genotypes (Bastille, Tunkahuan, 1_Canar, 5_Chimborazo), and corresponding to the four light environments. (R indicates the row, and C indicates the column).


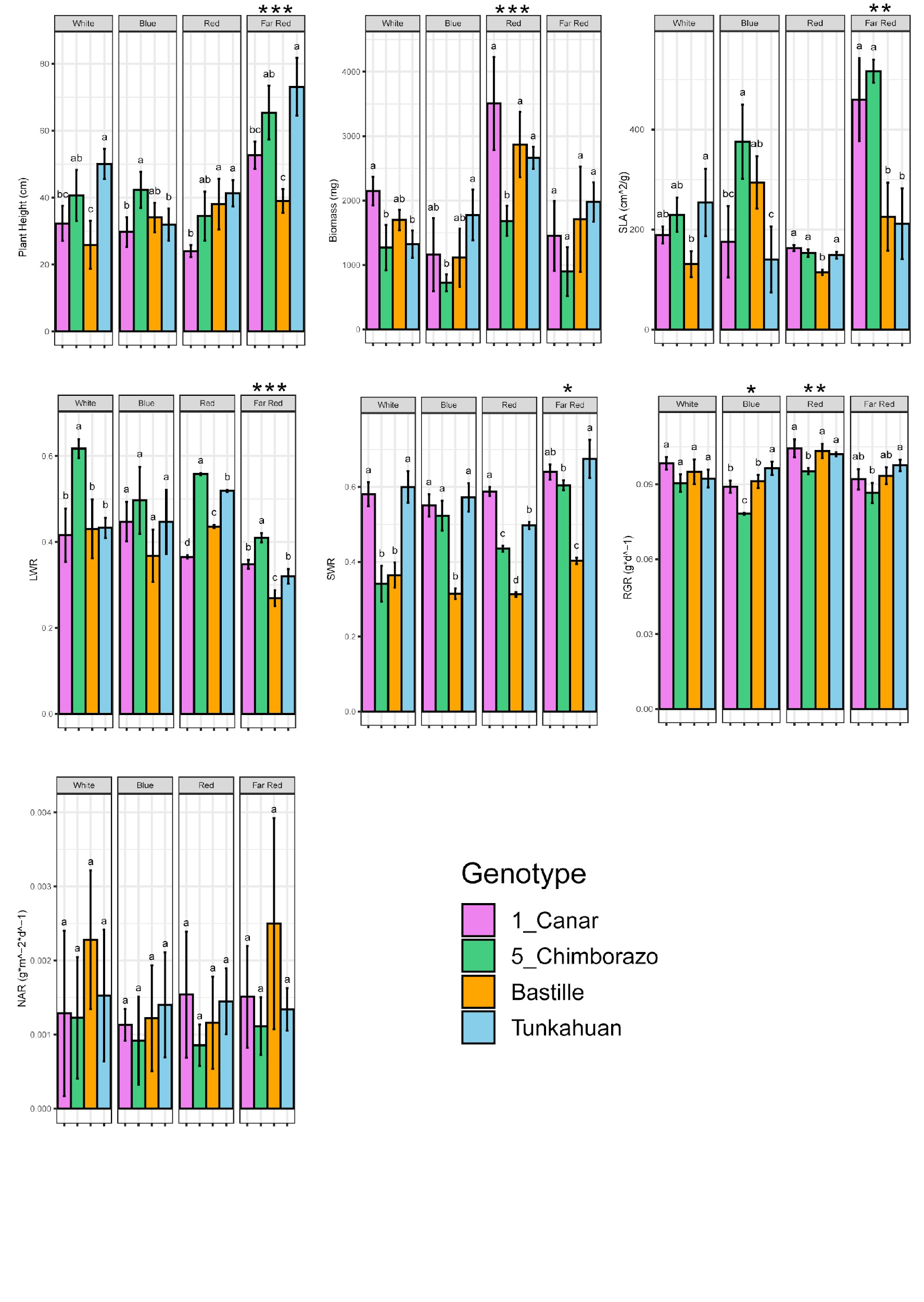


**Figure S3.** Plant traits analyzed under four light treatments (white control, blue, red, far-red) in the absence of salt in four quinoa genotypes. Traits were measured at week 12. a) Plant height, b) Total biomass c) Specific Leaf Area (SLA), d) Leaf Weight Ratio (LWR), e) Stem Weight Ratio (SWR), f) Relative Growth Rate (RGR), and g) Net Assimilation Rate (NAR). According to multiple-comparison tests, letters indicate differences among genotype means within each light treatment, while asterisks indicate differences among light treatments relative to the white control. Significance levels for comparisons between light treatments: * p ≤ 0.05, ** p ≤ 0.01, *** p ≤ 0.001.


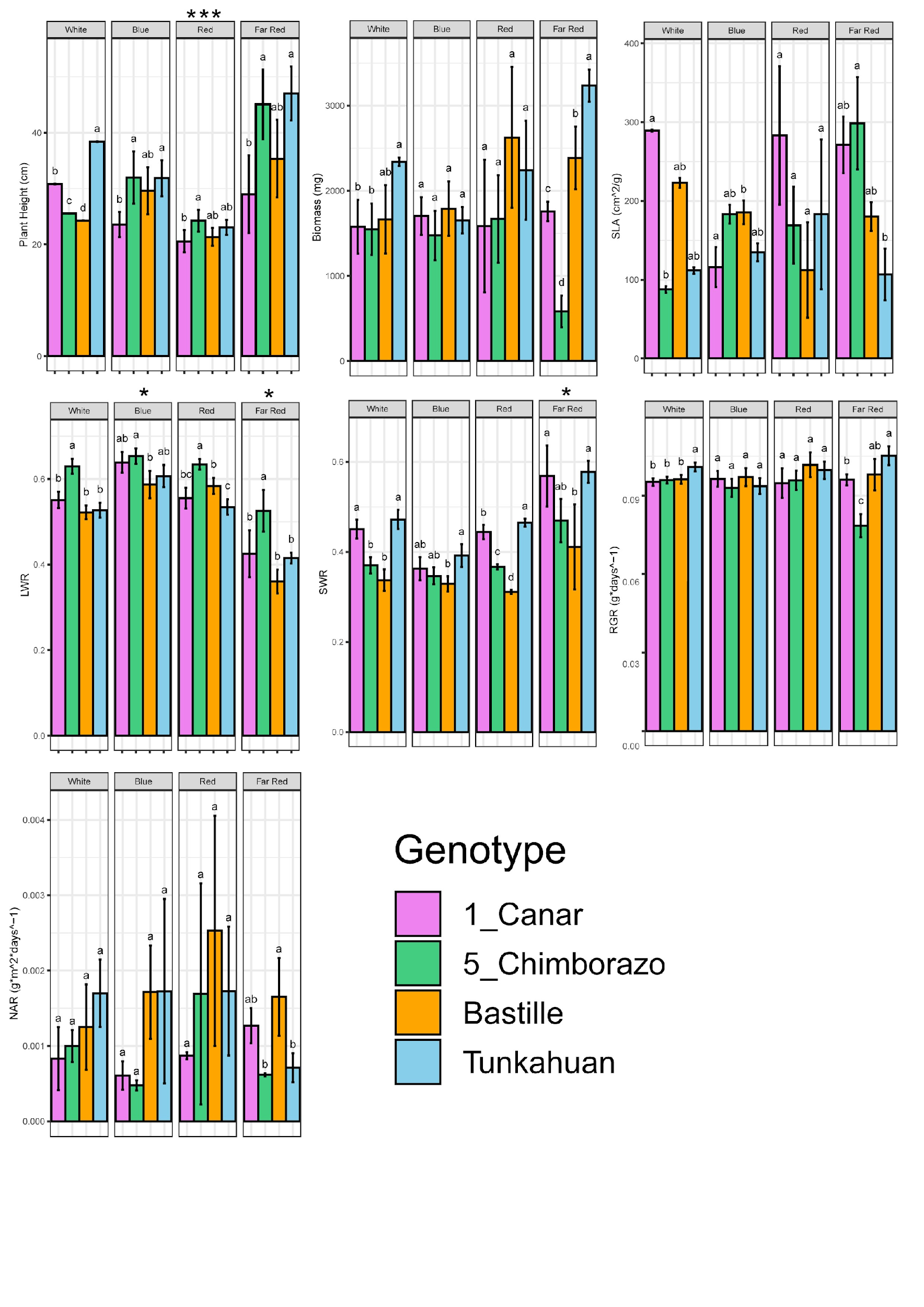


**Figure S4.** Plant traits analyzed under four light treatments (control white, blue, red, far-red) and a moderate salt concentration (200 mM NaCl) in four quinoa genotypes. Traits were measured at week 12. a) Plant height, b) Total biomass c) Specific Leaf Area (SLA), d) Leaf Weight Ratio (LWR), e) Stem Weight Ratio (SWR), f) Relative Growth Rate (RGR), and g) Net Assimilation Rate (NAR). According to multiple-comparison tests, letters indicate differences among genotype means within each light treatment, while asterisks indicate differences among light treatments relative to the white control. Significance levels for comparisons between light treatments: * p ≤ 0.05, ** p ≤ 0.01, *** p ≤ 0.001.


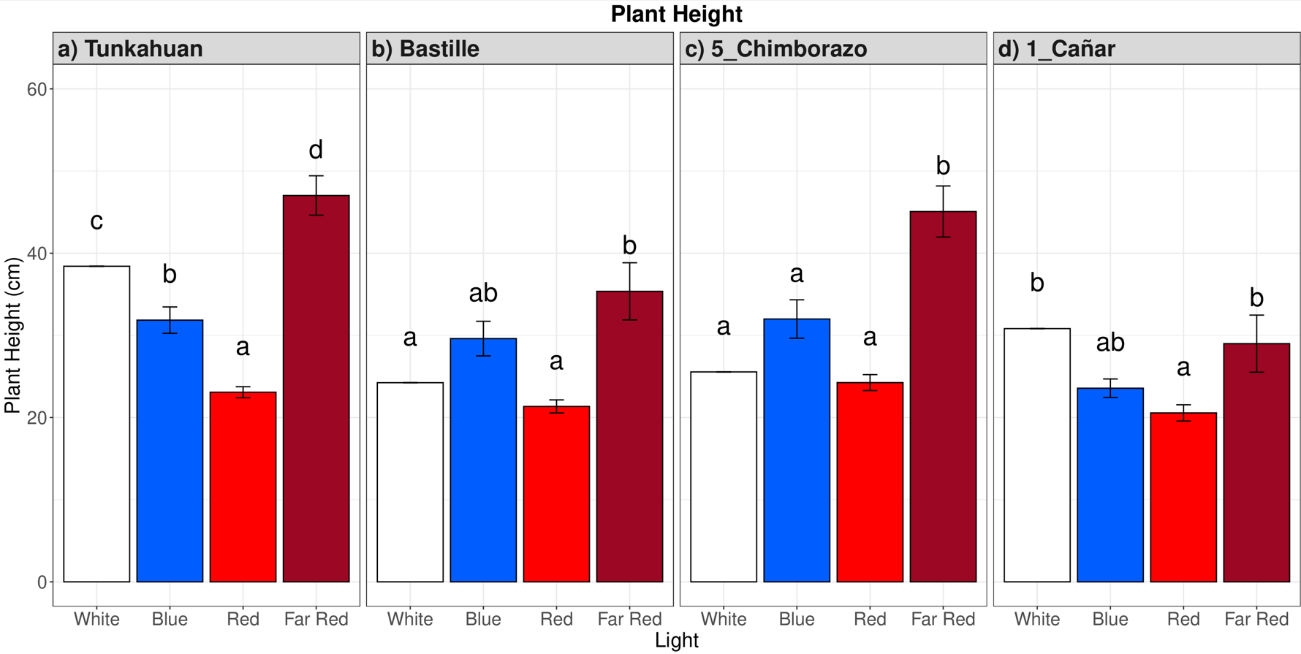


**Figure S5.** Plant height per genotype under four light treatments and salt treatment (200 mM NaCl). (a) Tunkahuan, (b) Bastille, (c) 5_Chimborazo, (d) 1_Cañar. Different letters indicate significantly different values after ANOVA and Tukey tests.


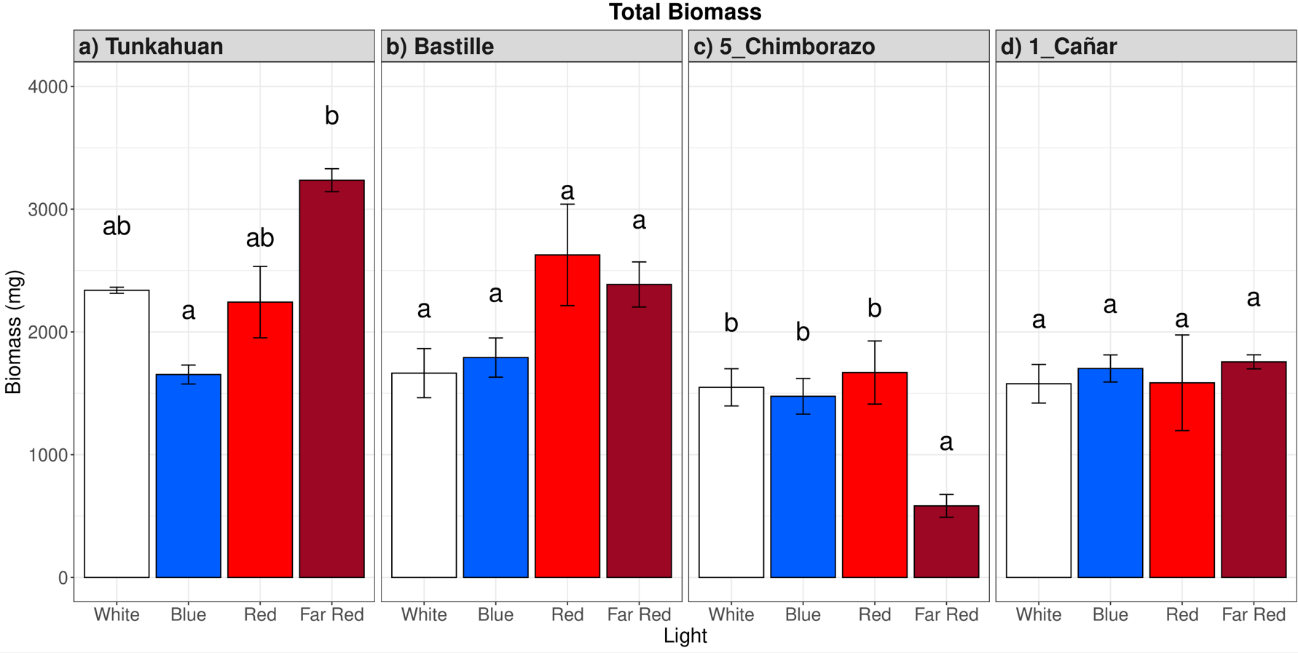


**Figure S6.** Total biomass per genotype under four light treatments and salt treatment (200 mM NaCl). (a) Tunkahuan, (b) Bastille, (c) 5_Chimborazo, (d) 1_Cañar. (Different letters indicate significantly different values after ANOVA and Tukey tests.


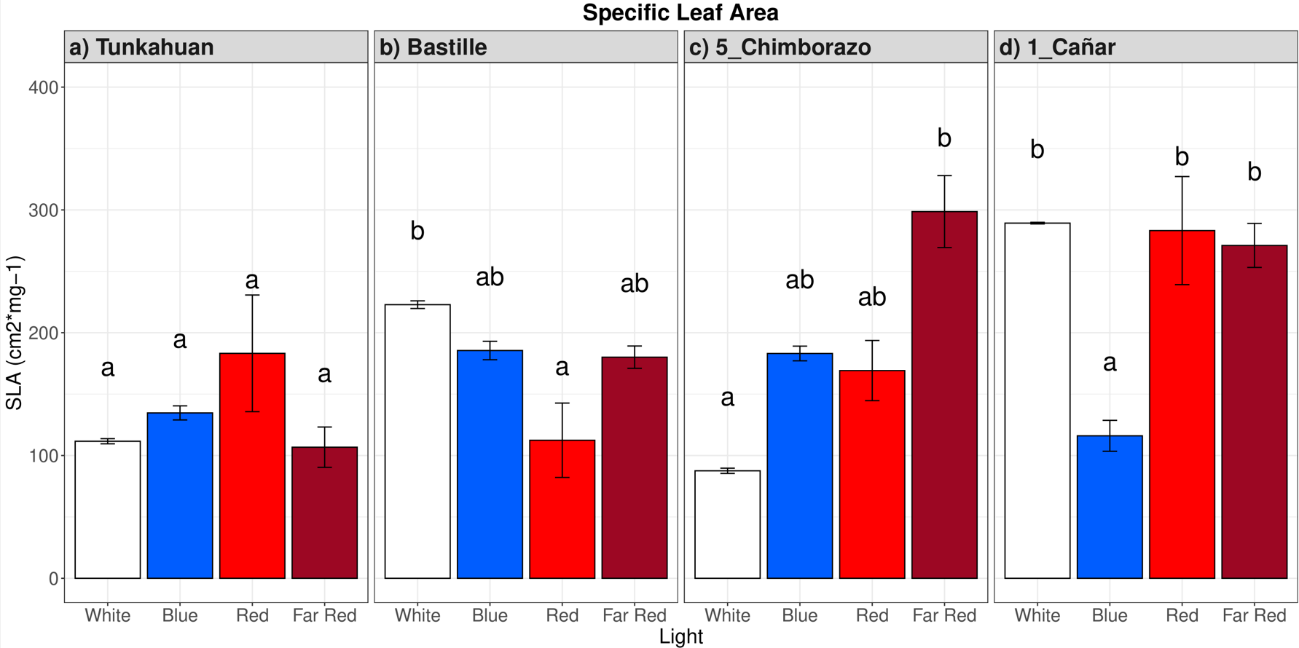


**Figure S7.** Specific Leaf Area (SLA) per genotype under four light treatments and salt treatment (200 mM NaCl). (a) Tunkahuan, (b) Bastille, (c) 5_Chimborazo, (d) 1_Cañar. Different letters indicate significantly different values after ANOVA and Tukey tests.


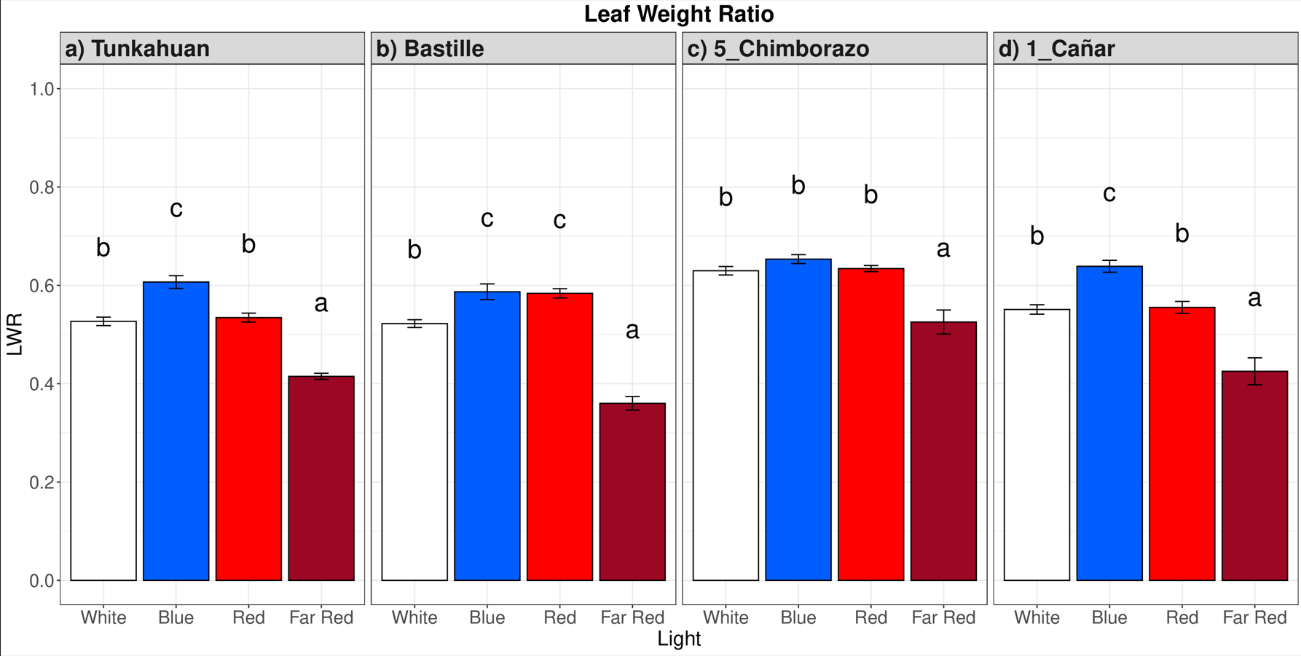


**Figure S8.** Leaf Weight Ratio (LWR) per genotype under four light treatments and salt treatment (200 mM NaCl). (a) Tunkahuan, (b) Bastille, (c) 5_Chimborazo, (d) 1_Cañar. Different letters indicate significantly different values after ANOVA and Tukey tests.


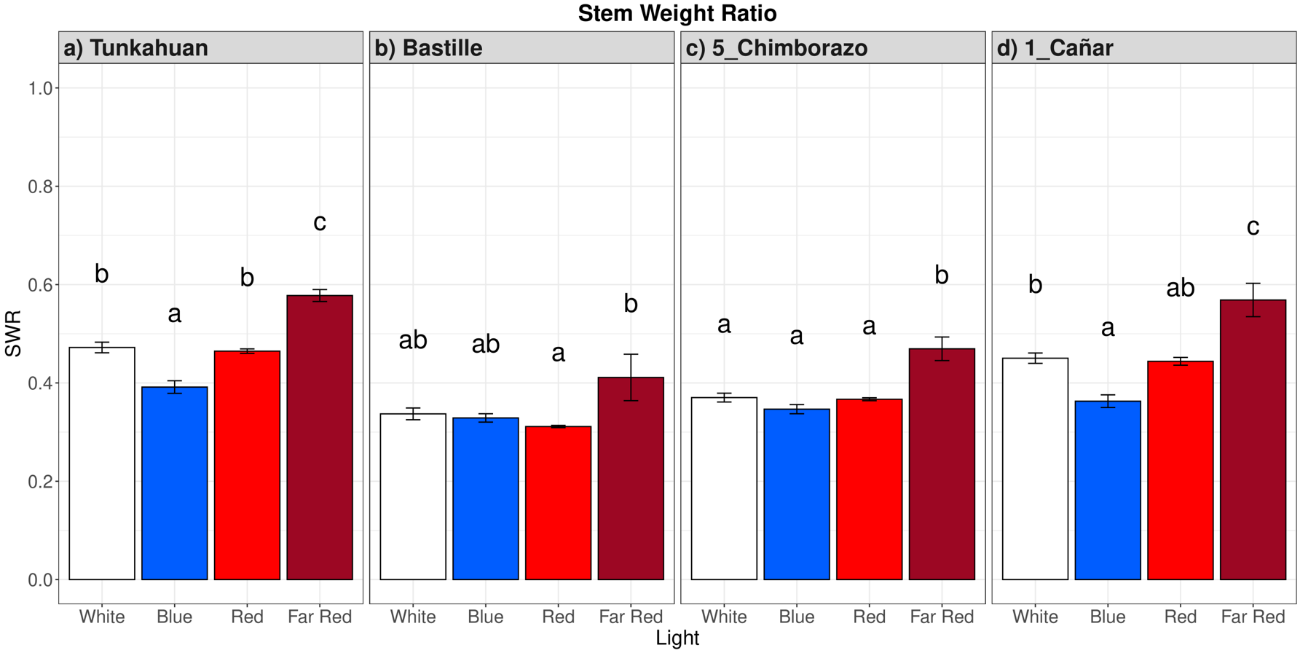


**Figure S9.** Stem Weight Ratio (SWR) per genotype under four light treatments and salt treatment (200 mM NaCl) (a) Tunkahuan, (b) Bastille, (c) 5_Chimborazo, (d) 1_Cañar. Different letters indicate significantly different values after ANOVA and Tukey tests.


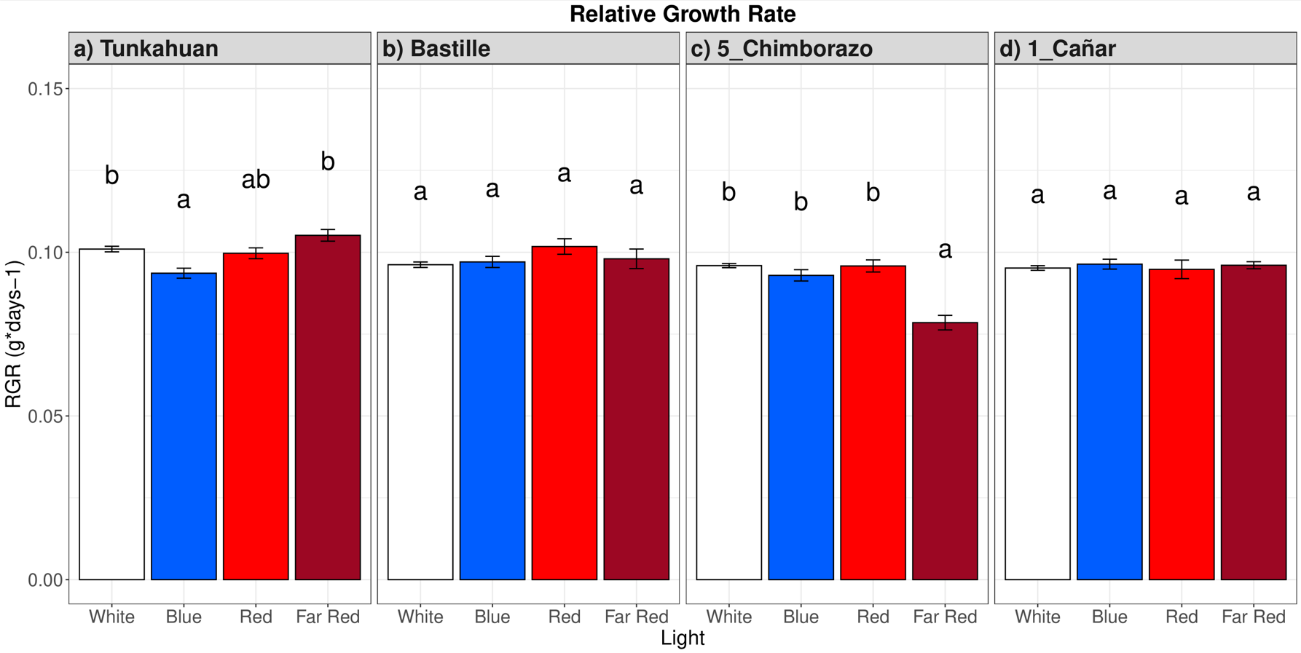


**Figure S10.** Relative Growth Rate (RGR) per genotype under four light treatments and salt treatment (200 mM NaCl). (a) Tunkahuan, (b) Bastille, (c) 5_Chimborazo, (d) 1_Cañar. Different letters indicate significantly different values after ANOVA and Tukey tests.


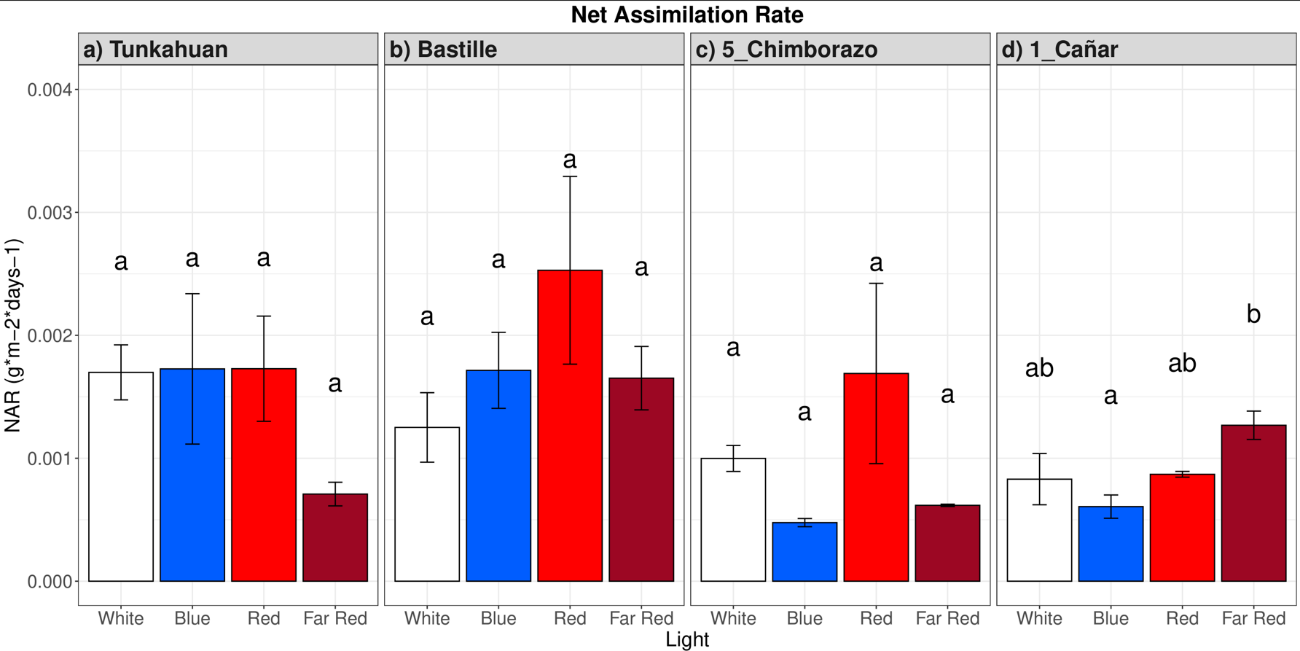


**Figure S11.** Net Assimilation Rate (NAR) per genotype under four light treatments and salt treatment (200 Mm NaCl). (a) Tunkahuan, (b) Bastille, (c) 5_Chimborazo, (d) 1_Cañar. Different letters indicate significantly different values after ANOVA and Tukey tests.


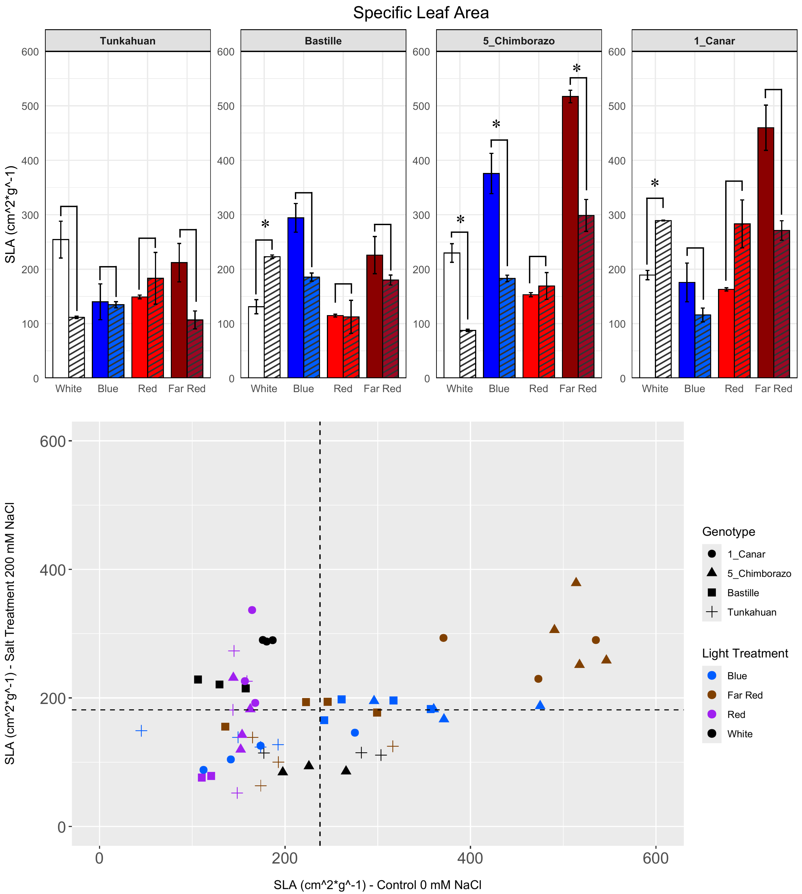


**Figure S12.** Effect of salt on Specific Leaf Area (SLA) per genotype under four light treatments. (a) Tunkahuan, (b) Bastille, (c) 5_Chimborazo, (d) 1_Cañar under the absence and presence of salt and under white and supplementary blue, red, and far-red light. Values obtained under control and salinity conditions were compared using Student's t-test or Kruskal-Wallis test, depending on data normality. Asterisks indicate statistically significant differences between control and treatment. (e) Two-dimensional scatterplot. Each point represents SLA under a specific light treatment (shown by color) and by genotype (shown by symbol). The x-axis represents absence of salt, the y-axis presence of salt. Dashed vertical and horizontal lines indicate the overall mean for control and salt conditions, respectively. The dashed lines the plot into four quadrants: (1) upper right—plants performing above average in both conditions, that is, under the absence and presence of salt; (2) upper left—plants performing better under salt than under no salt; (3) lower right—plants performing better under no salt than under salt; and (4) lower left—plants performing below average in both the absence and the presence of salt.


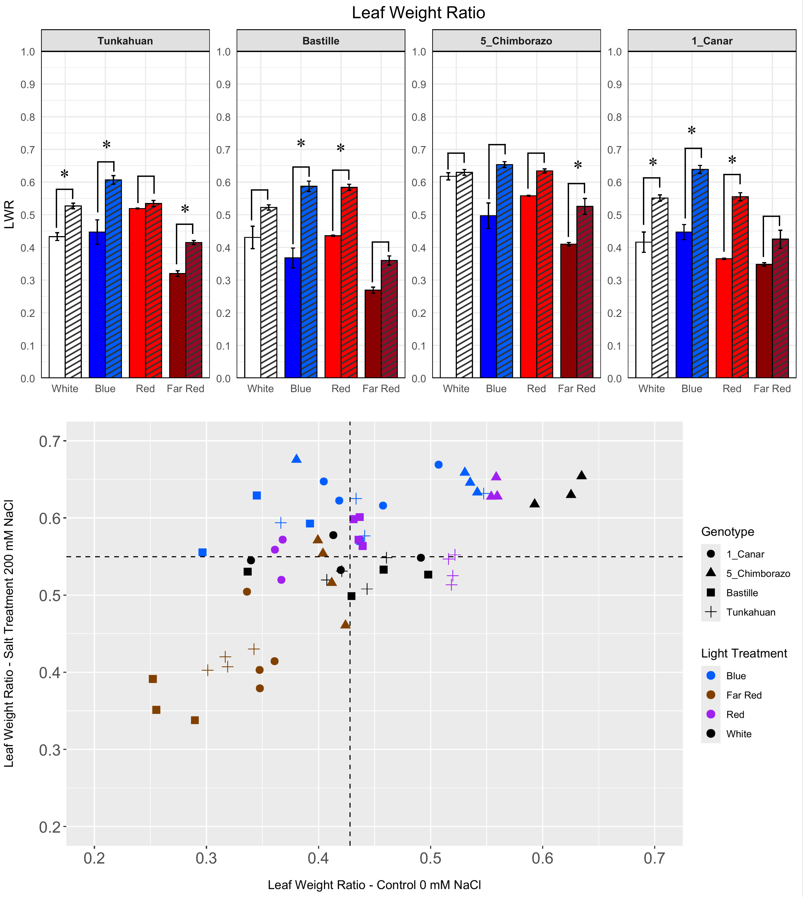


**Figure S13.** Effect of salt on Leaf Weight Ratio (LWR) per genotype under four light treatments. (a) Tunkahuan, (b) Bastille, (c) 5_Chimborazo, (d) 1_Cañar under the absence and presence of salt and under white and supplementary blue, red, and far-red light. Values obtained under control and salinity conditions were compared using Student's t-test or Kruskal-Wallis test, depending on data normality. Asterisks indicate statistically significant differences between control and treatment. (e) Two-dimensional scatterplot. Each point represents LWR under a specific light treatment (shown by color) and by genotype (shown by symbol). The x-axis represents absence of salt, the y-axis presence of salt. Dashed vertical and horizontal lines indicate the overall mean for control and salt conditions, respectively. The dashed lines the plot into four quadrants: (1) upper right—plants performing above average in both conditions, that is, under the absence and presence of salt; (2) upper left—plants performing better under salt than under no salt; (3) lower right—plants performing better under no salt than under salt; and (4) lower left—plants performing below average in both the absence and the presence of salt.


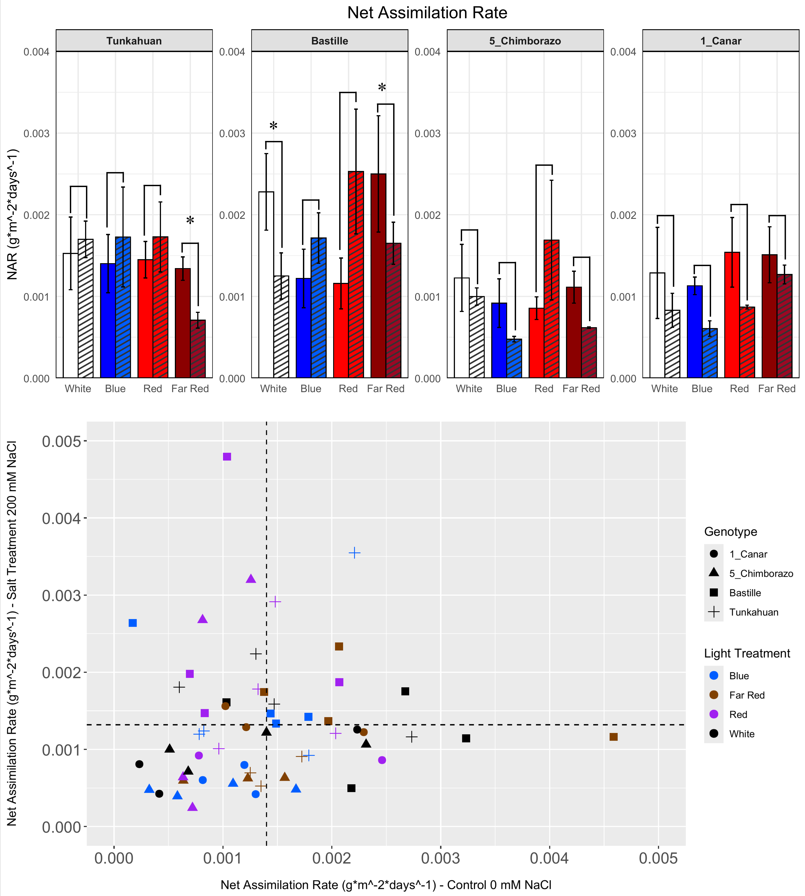


**Figure S14.** Effect of salt on Net Assimilation Rate (NAR) per genotype under four light treatments. (a) Tunkahuan, (b) Bastille, (c) 5_Chimborazo, (d) 1_Cañar under the absence and presence of salt and under white and supplementary blue, red, and far-red light. Values obtained under control and salinity conditions were compared using Student's t-test or Kruskal-Wallis test, depending on data normality. Asterisks indicate statistically significant differences between control and treatment. (e) Two-dimensional scatterplot. Each point represents NAR under a specific light treatment (shown by color) and by genotype (shown by symbol). The x-axis represents absence of salt, the y-axis presence of salt. Dashed vertical and horizontal lines indicate the overall mean for control and salt conditions, respectively. The dashed lines the plot into four quadrants: (1) upper right—plants performing above average in both conditions, that is, under the absence and presence of salt; (2) upper left—plants performing better under salt than under no salt; (3) lower right—plants performing better under no salt than under salt; and (4) lower left—plants performing below average in both the absence and the presence of salt.


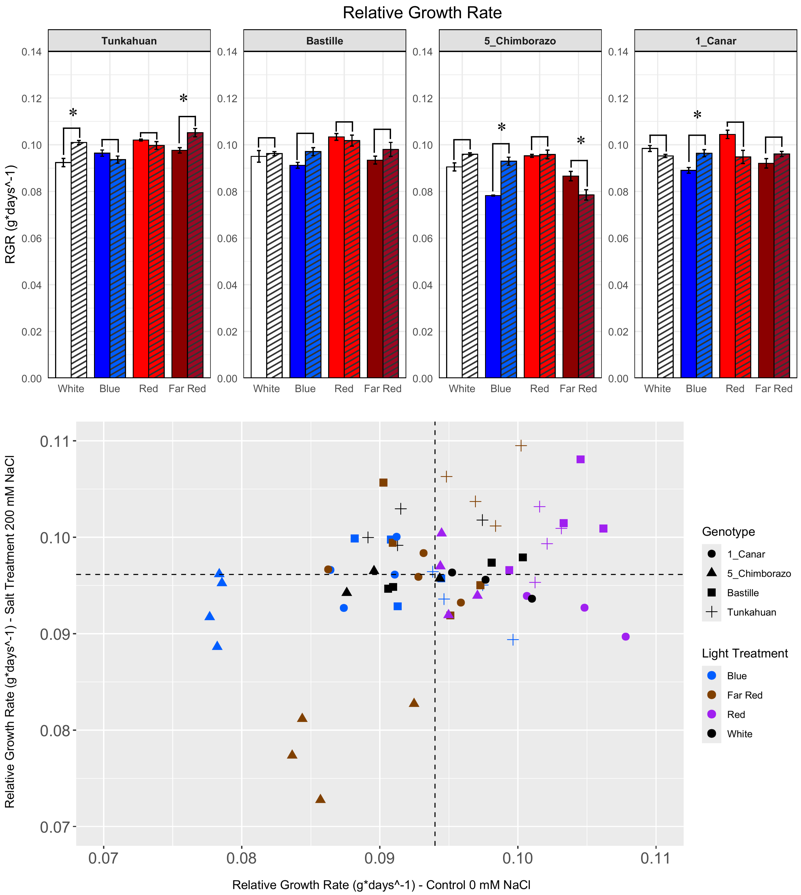


**Figure S15.** Effect of salt on Relative Growth Rate (RGR) per genotype under four light treatments. (a) Tunkahuan, (b) Bastille, (c) 5_Chimborazo, (d) 1_Cañar under the absence and presence of salt and under white and supplementary blue, red, and far-red light. Values obtained under control and salinity conditions were compared using Student's t-test or Kruskal-Wallis test, depending on data normality. Asterisks indicate statistically significant differences between control and treatment. (e) Two-dimensional scatterplot. Each point represents RGR under a specific light treatment (shown by color) and by genotype (shown by symbol). The x-axis represents absence of salt, the y-axis presence of salt. Dashed vertical and horizontal lines indicate the overall mean for control and salt conditions, respectively. The dashed lines the plot into four quadrants: (1) upper right—plants performing above average in both conditions, that is, under the absence and presence of salt; (2) upper left—plants performing better under salt than under no salt; (3) lower right—plants performing better under no salt than under salt; and (4) lower left—plants performing below average in both the absence and the presence of salt.
